# Improving agricultural spraying with multi-rotor drones: a technical study on operational parameter optimization

**DOI:** 10.3389/fnut.2024.1487074

**Published:** 2024-12-18

**Authors:** D. Yallappa, R. Kavitha, A. Surendrakumar, B. Suthakar, A. P. Mohan Kumar, Balaji Kannan, M. K. Kalarani

**Affiliations:** ^1^Department of Farm Machinery and Power Engineering, Agricultural Engineering College and Research Institute, Tamil Nadu Agricultural University, Coimbatore, India; ^2^Department of Soil and Water Conservation Engineering, Agricultural Engineering College and Research Institute, Tamil Nadu Agricultural University, Coimbatore, India; ^3^Directorate of Crop Management, Tamil Nadu Agricultural University, Coimbatore, India

**Keywords:** boom spray, discharge rate, drone sprayer, distribution pattern, hover height, patternator

## Abstract

Drones play a key role in enhancing nutrient management efficiency under climate change scenarios by enabling precise and adaptable spray applications. Current aerial spray application research is primarily focused on examining the influence of drone spraying parameters *viz.,* flight height, travel speed, rotor configuration, droplet size, payload, spray pressure, spray discharge and wind velocity on spray droplet deposition characteristics. The present study aimed to study and optimize the effect of spray height, operating pressure, nozzle spacing and spray nozzle mounting configuration on spray discharge rate, spray width, spray distribution pattern, spray uniformity and spray liquid loss. A spray patternator of 5.0 m x 5.0 m was developed per Bureau of Indian Standards (BIS) standard to study the spray volume distribution pattern of boom and hex nozzle configuration. Initially, drone spray operational parameters *viz.,* spray discharge rate (Lm^−1^), operating pressure (kg cm^−2^) and spray angle (°) were measured using digital nozzle tester, digital pressure gauge and digital protractor, respectively, in the laboratory. Then optimized the nozzle spacing for boom configuration attachment to drone sprayer and recorded best spray uniformity at 0.6 m nozzle spacing. The drone sprayer hovered at three different heights, *viz.,* 1.0, 2.0 and 3.0 m from the top of the patternator and spray operating pressure was maintained at 4.0 kg cm^−2^ in outdoor condition. Single pass distribution pattern and one-direction application distribution pattern method used for optimizing height of spray, operating pressure and nozzle mounting confirmation from the results of discharge rate, spray angle, effective spray width, spray liquid loss and spray distribution uniformity. Results showed that, the better spray uniformity distribution was found when the drone sprayer hover height was increased from the top of the patternator (2.0 m). More round spray droplet vertex pattern was generated during the 1.0 m hover height compared to the 2.0 and 3.0 m hover heights due to the direct impact of downwash airflow generated by the rotors. Finally it was concluded that, the good spray volume distribution was found at 2.0 m height of spray with standard hexa nozzle configuration arrangement as compared to the boom spray nozzle arrangement.

## 1 Introduction

Technological advancements in precision farming, especially through the integration of multi-rotor drones with sprayer devices have revolutionized agricultural practices. These drones enable precise and targeted nutrient management, enhancing crop efficiency and resilience in the face of climate change, thus have transformed pesticide, herbicide, and fertilizer applications (1, 2). These drones provide precise, targeted pesticide delivery, reducing waste, lowering environmental impact, and boosting overall crop health (3). The efficiency of these drone sprayers, however, is strongly reliant on the optimization of their operational parameters. Spray height, nozzle type, droplet size, flight speed, and application rate must all be precisely adjusted to guarantee even coverage and successful pest control. Optimizing these parameters is crucial not only for maximizing the efficacy of the chemicals used, but also for preserving and improving crop nutritional quality. Drones have greatly become benefited with advanced features in autonomous spraying systems, including autonomous path planning, break point continue to spray (4), terrain following radar module (auto altitude adjustment), high-precision obstacle avoidance radar, spray task list, spray solution empty indication, battery level warning, and high-accuracy Real Time Kinematics (RTK) location to significantly increase functional stability, efficiency, accuracy, and ease of use (5). Nutrient composition is directly influenced by the right executive management of nutrients and protective agents, which aids in crop development (6, 7). For example, uniform and optimal spraying can guarantee that crops receive the proper amount of nutrients, resulting in improved growth and nutrient buildup in edible sections of the plant. Furthermore, accurate spraying can reduce crop stress from pests, diseases, and environmental conditions, increasing their resistance. Stress resilience, another important element of crop health, can be strongly influenced by the efficacy of pesticide use. Crops that are less stressed grow faster, produce more, and have superior nutritional profiles. The drone machine operational parameters, *viz.*, flight height, travel speed, payload and configuration, have a great impact on the distribution and penetration of droplets (8). The most important benefit of using a drone (multi-rotor) for chemical spraying is that, due to its unique rotor structure and principle of motion, it generates powerful downwash airflow during flight operation, changing the crop disturbance and improving liquid penetration (9). The downwash airflow velocity of the rotors can create a strong velocity distribution of plants during spraying. This helps the spray droplets to atomize much further with enhanced deposition onto the crop surface. Spray droplet velocity has positive effects on spray swath, deposition, and drift, influencing the operation’s consequences (10). Yallappa et al. (11) studied spray volume distribution pattern for boom nozzle configuration using drone sprayer under laboratory condition.

There is lack of detailed study regarding performance of spray operational parameters *viz.*, height of spray, spray pressure, travel speed, discharge rate, spray droplet distribution uniformity and spray nozzle spacing for efficient chemical spray application using drone sprayer. Commercial drone manufacturers are adopting drones without having basic information on the performance and efficacy of the drone spraying system in terms height of spray, nozzle flow rate, operating pressure, type of nozzle and nozzle configuration, spray uniformity and application rate. The present investigation was taken up to study and optimize spray operational parameters of drone sprayer under laboratory condition.

The overall goal of this research was to develop a suitable size patternator for measuring, analyzing and optimizing the spray operational parameters of drone sprayer with the specific objectives *viz.*, (1) development of a suitable customized spray patternator for measuring spray pattern distributions; (2) evaluation for optimizing the effect of height of spray, nozzle spacing and operating pressure on spray discharge rate, effective spray width, spray angle, spray uniformity and spray volume distribution pattern (3) investigation on the impact of drone sprayer machine parameter as downwash airflow on spray distribution systems at different hover heights in outdoor conditions. (4) Recommending the best spray nozzle configuration to drone sprayer based on the spray volume uniformity distribution before actual use in field condition. Optimizing the spray operational parameters of multi-rotor agricultural drones is crucial for accurately estimating and enhancing crop resilience. By delivering precise, timely, and efficient applications, these drones support the development of stronger, more resilient crops, ultimately contributing to sustainable agricultural practices and improved food and nutritional security.

## 2 Materials and methods

### 2.1 Drone

**Figure 1:**
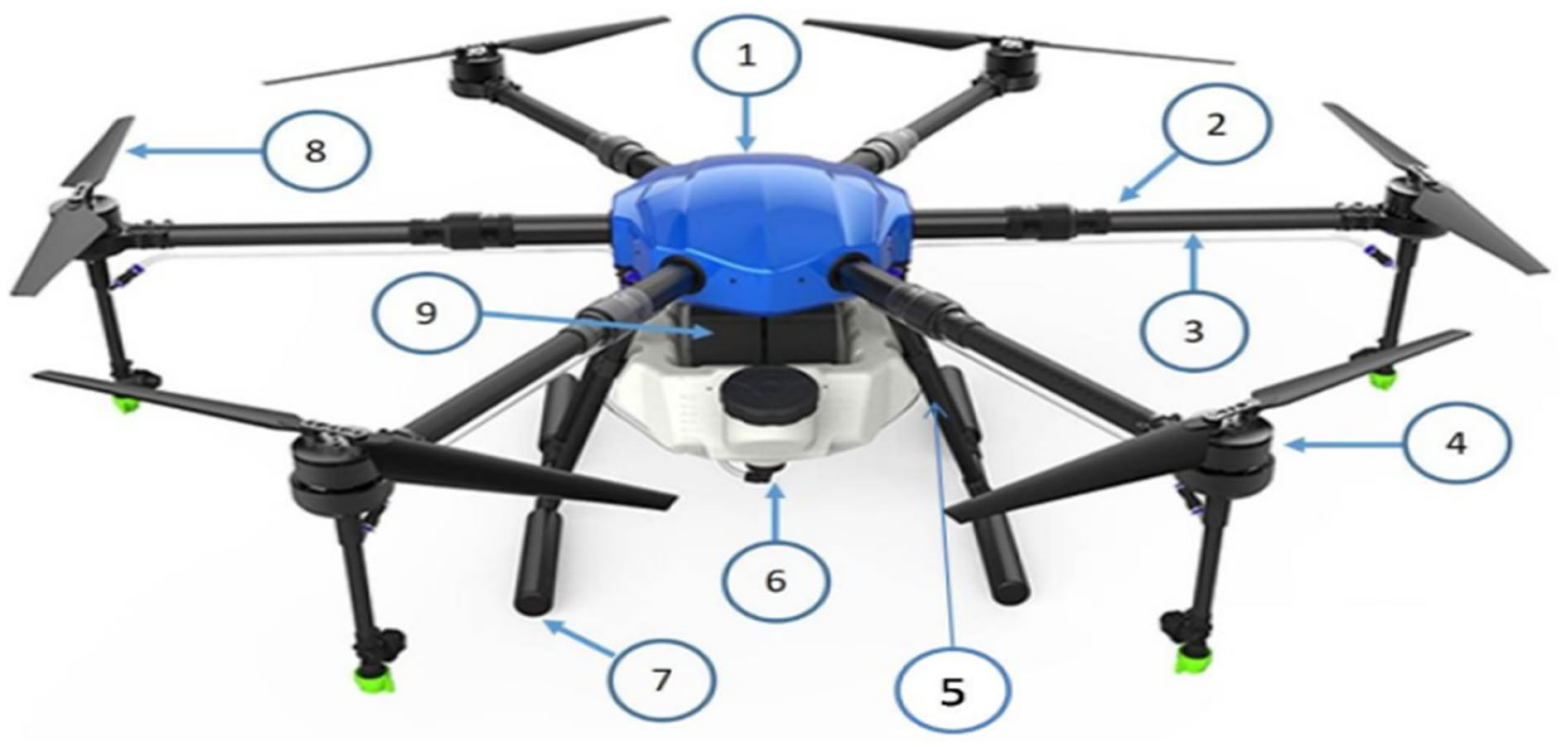
Electric battery-operated drone sprayer.

The drone sprayer used in the present investigation, was an E610P six-rotor electric (M/s. EFT Electronic Technology Co., Ltd., Hefei City, China) is shown in Figure 1 and specifications are presented in Table 1. The spraying system of the drone sprayer mainly consisted of Flight controller (1), Brushless direct current (BLDC) motors arm (2), Fluid hose pipe (3), BLDC motor (4), Support frame (5), Pesticide tank (6), Landing gear (7), Foldable propeller (8), Lithium polymer (LiPo) batteries (9). The UAV sprayer has two LiPo batteries of 6 cells each with a capacity of 16,000 mAh to supply the necessary current required for the propulsion system. A 24 V BLDC motor coupled with a pump was used to pressurize the spray liquid and then atomize it into fine spray droplets. This drone spray model has the functions of GPS route planning and breakpoint return, which could complete aerial spraying operations autonomously.

**Table 1:** Specifications of drone sprayer.

Main parameter	Norms and numerical values
Type	Hexacopter
Item Model	E610P
Unfold fuselage size, (L × W × H), mm	2000 × 1800 × 670
Folding Size, (L × W × H), mm	950 × 850 × 670
Power source	12S 16,0000 mAh LiPo Battery
Payload capacity, L	10
Self-weight, kg	6.9
Take-off weight, kg	26
Flight height, m	1–20
Forward travel speed, ms^−1^	0–8
Type of spray nozzle	Flat fan shape
Number of nozzles	4
Discharge rate, l m^−1^	0–3.2
Swath width of spray, m	3–5
Liquid pressure, kg cm^−2^	3.4
Remote controller distance, km	1.5
No-load flight time, min	25
Charging time, min	90

### 2.2 Study the machine and operational parameters of drone sprayer

The selected autonomous battery-operated drone sprayer (Make: EFT Electronic Technology, Model: E610P) was tested and calibrated in the laboratory condition Agricultural Machinery Research Centre (AMRC), Department of Farm Machinery and Power Engineering, Agricultural Engineering College and Research Institute Agricultural University, Coimbatore (11.0122° N, 76.9354° E) by taking different variables which mainly influence the functional performance. ASAE (S341.5) standard calibration procedure has been followed to assess the different spray operational parameters (19).

#### 2.2.1 Measurement of nozzle discharge rate, spray operating pressure and spray angle

A handheld portable digital nozzle tester (AAMS, Maldegem, Belgium) and digital liquid pressure gauge instrument (Make: Shanghai XuYan Precision Technology Co., Ltd., Model: XY-PG560R) were used to measure spray liquid discharge rate and operating pressure at specific time interval while operating the drone sprayer under normal conditions. A24 V DC brushless direct current (BLDC) motor and diaphragm pump were used to pressurize the spray fluid. This BLDC motor is connected to power distribution board and signal wires were connected to motor port in flight controller (Make: JIYI, Model: K++ V2, Version: V1.5.1).

**Figure 2:**
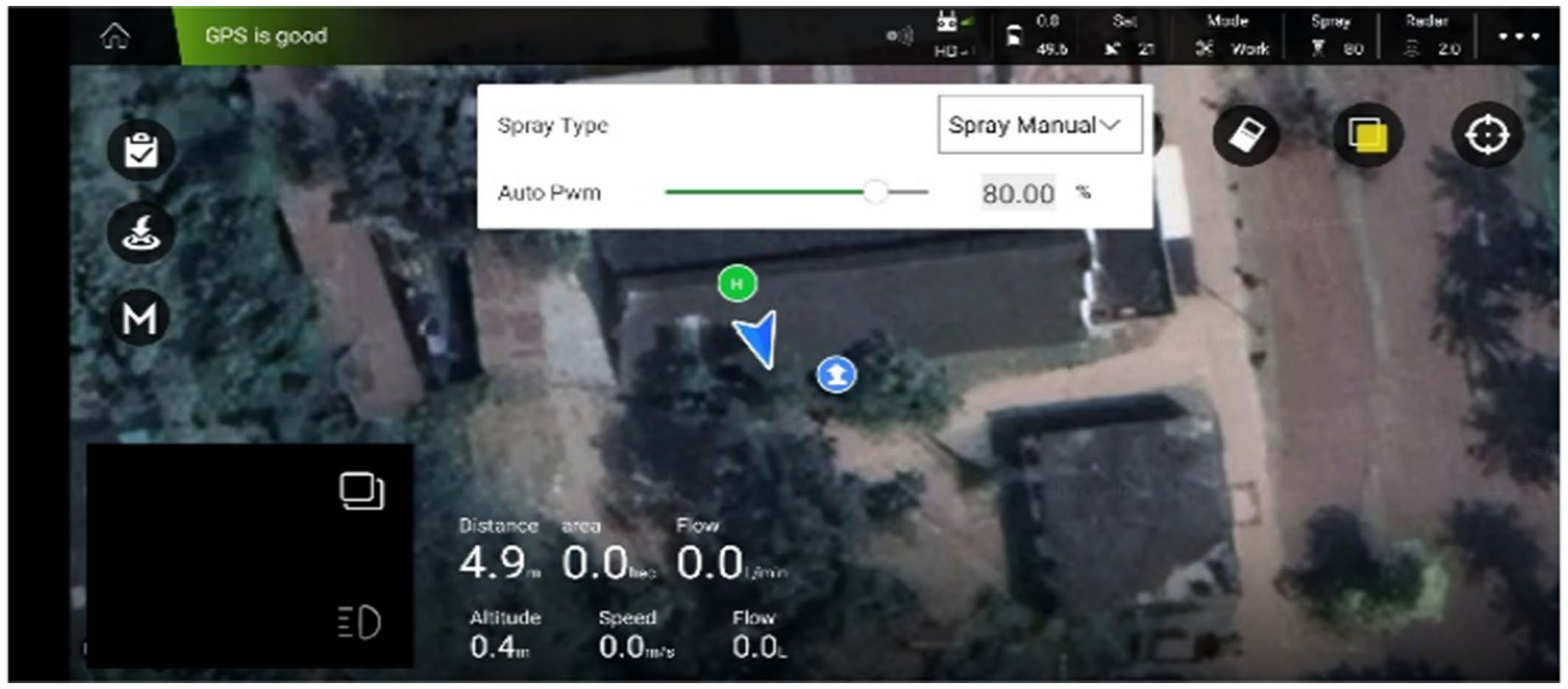
Screen display view of intelligent/precise spray liquid flow control system in terms of PWM (%).

This autonomous drone sprayer has inbuilt intelligent/ precise spray discharge rate control system at different rotation of BLDC motor speed with PWM from Agri Assistant mobile app (Version: V1.5.1). The Agri Assistant App has a display showing the flow rate of diaphragm pump in terms of Pulse Width Modulation (PWM) ranging from 0 to 100% as shown in Figure 2.

#### 2.2.2 Experimental setup

**Figure 3:**
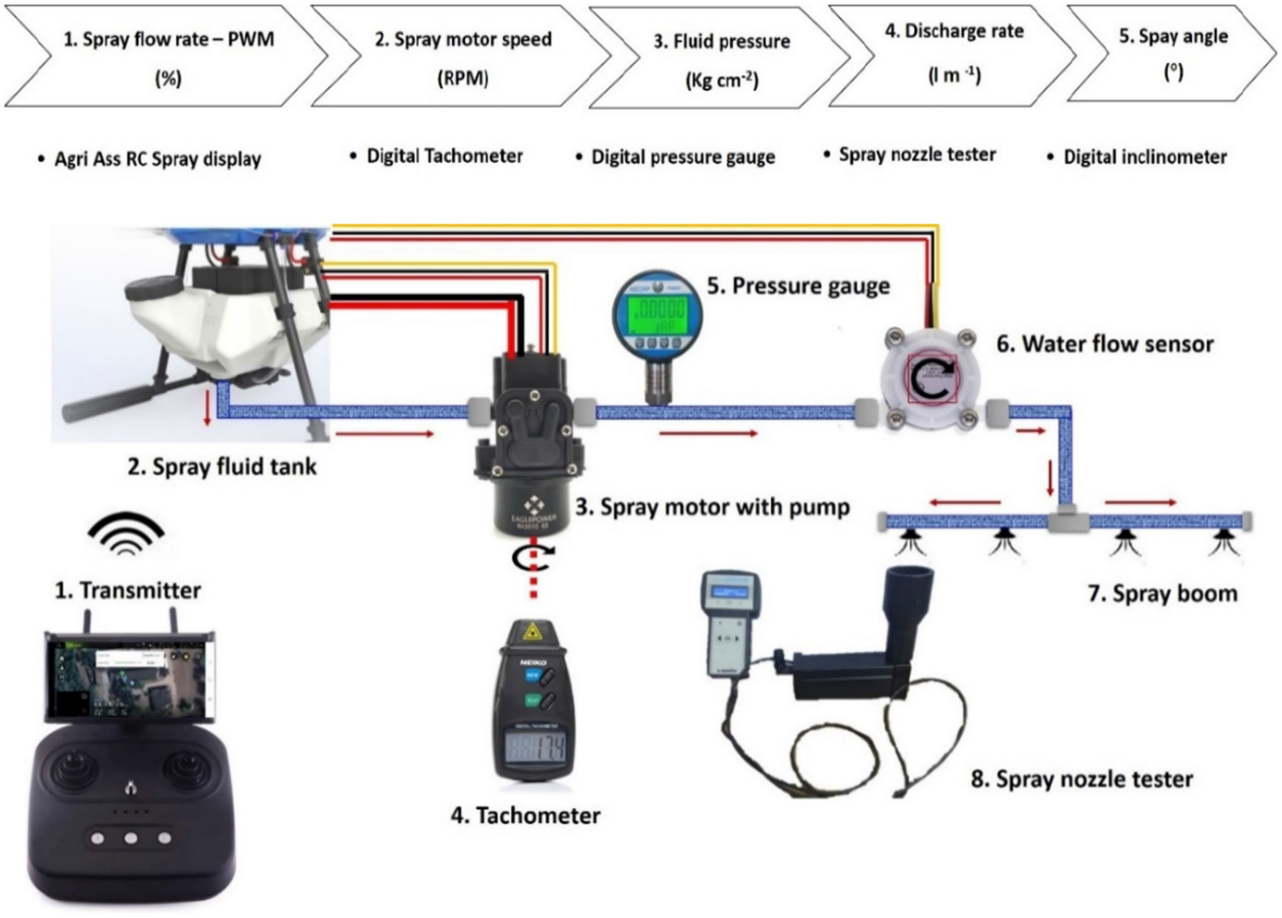
Experimental layout for measurement of spray motor speed, sprayer discharge rate, operating pressure and spray nozzle angle.

The diaphragm pump inlet is connected to the fluid tank and its outlet is connected to the main line of four nozzles (2020A-132 series, M/s Ningbo Licheng Agricultural Spray Technology Co., Ltd., Zhejiang, China). Digital liquid pressure gauge and spray flow sensor (Make: Sea, Model: T1A/K3A) were connected in between diaphragm pump outlet and nozzles main hose pipe and pressure was recorded in terms of kg cm^−2^. An experiment was conducted to assess the effect of drone sprayer BLDC spray motor speed on pump operating pressure, nozzle discharge rate and spray angle. The experimental layout in Agricultural Machinery Research Centre (AMRC), Department of Farm Machinery and Power Engineering, Agricultural Engineering College and Research Institute, Agricultural University, Coimbatore (11.0122° N, 76.9354° E) and is shown in Figure 3.

**Figure 4:**
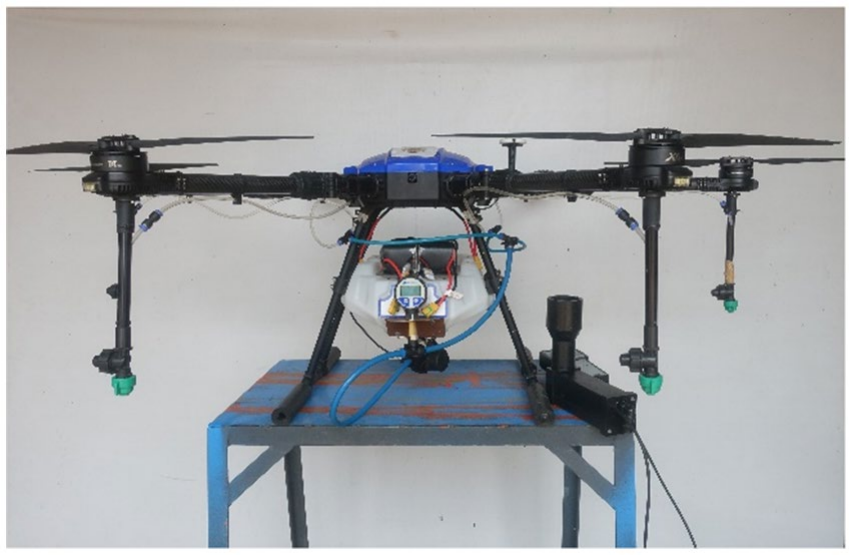
Experimental layout for measurement of spray discharge rate using nozzle tester.

**Figure 5:**
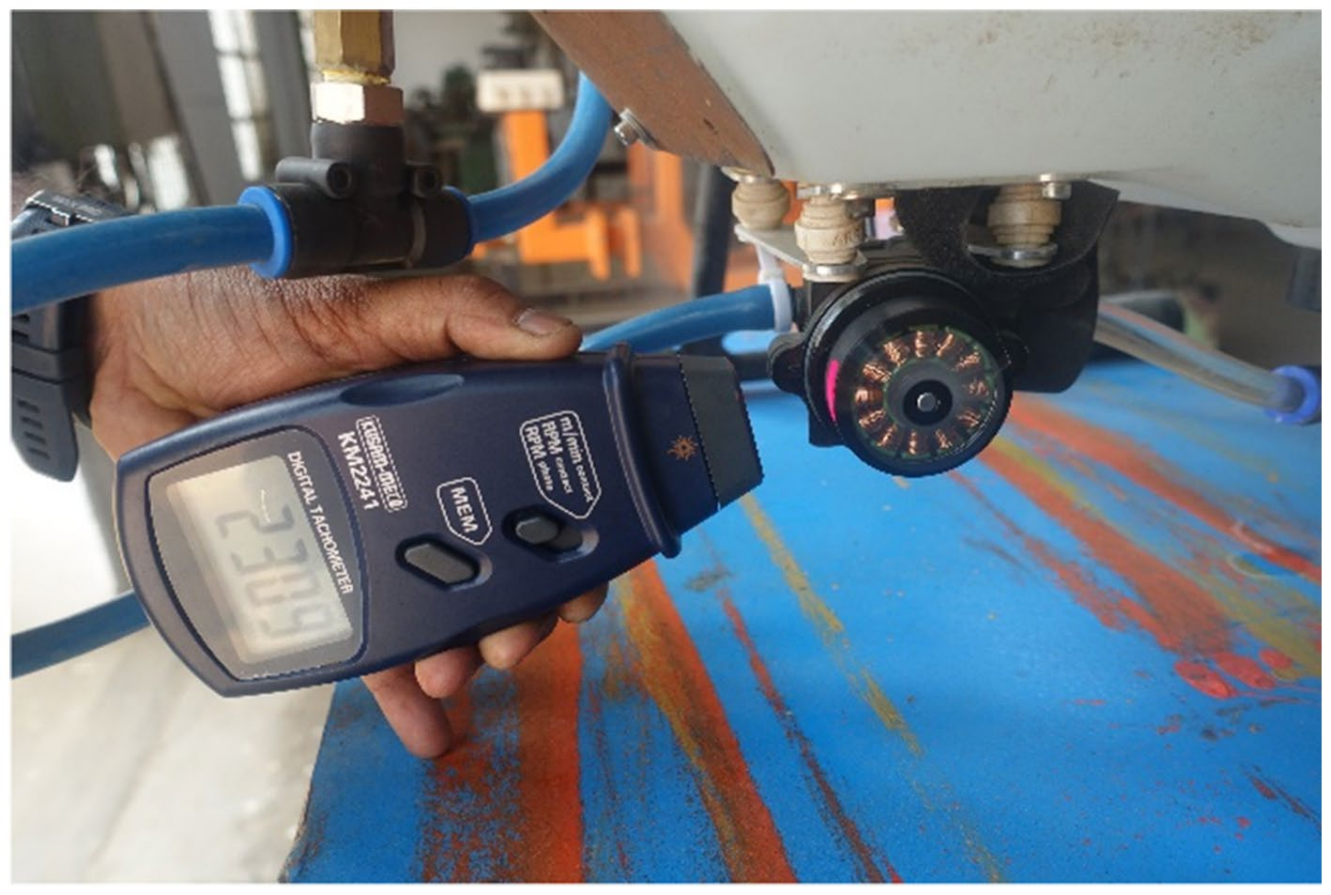
Experimental layout for measurement of spray motor speed.

**Figure 6:**
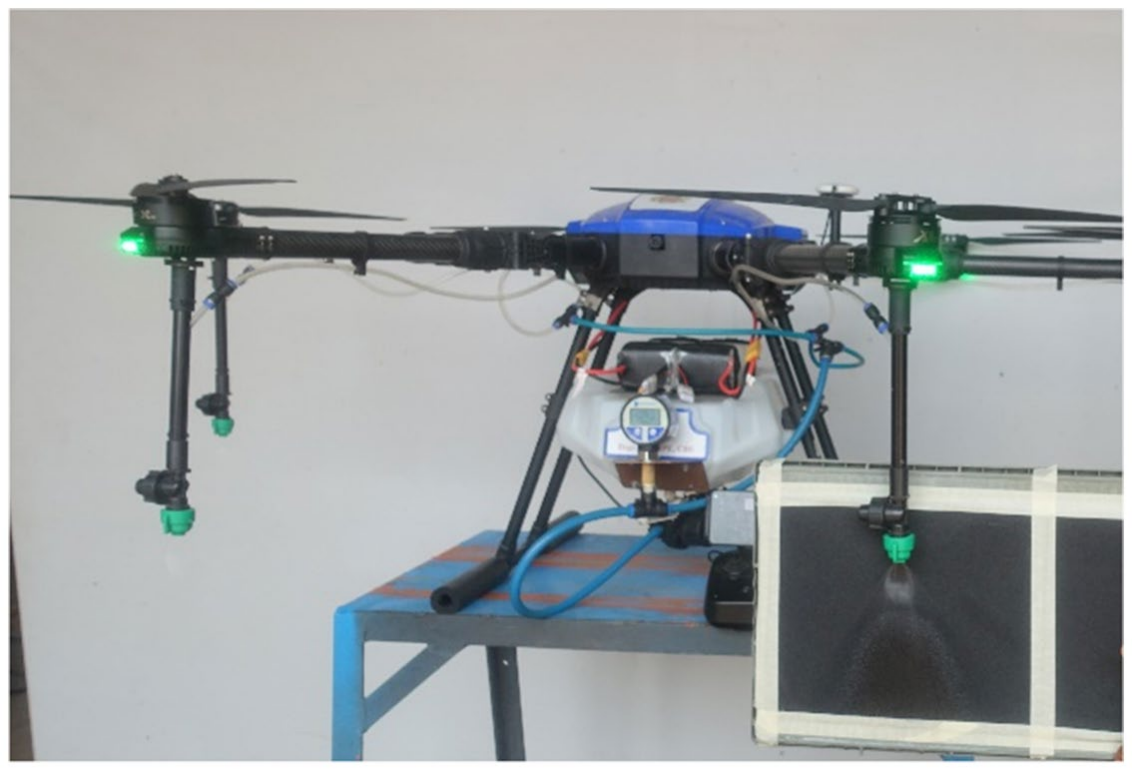
Experimental layout for measurement of spray angle.

Initially, transmitter sends the PWM percentage signals (1) to flight controller through receiver. Then spray water flows from fluid tank (2) to spray diaphragm pump (3). The rotational speed of BLDC motor was recorded as a PWM percentage and then converted it in to revolutions per minute using a contactless digital tachometer (Make: Kusam Meco, Model: Km-2234Bl) (4). The operating pressure of spray fluid was recorded using digital liquid pressure gauge instrument (5) and provides the live water flow rate feedback information from water flow sensor (6). Then, recorded the pump flow rate of nozzle (7) in terms of liter per min at 0, 10, 20, 30, 40, 50, 60, 70, 80, 90 and 100% of PWM using a handheld portable nozzle tester (8). Simultaneously, captured the images of each nozzle spray and calculated the actual spray angle using a digital protractor (Make: Yuzuki, Model: IP65). The experimental setup and arrangement for measurement of spray liquid discharge rate, spray motor speed and spray angle are shown in Figures 4–6, respectively.

### 2.3 Development of spray patternator

**Figure 7:**
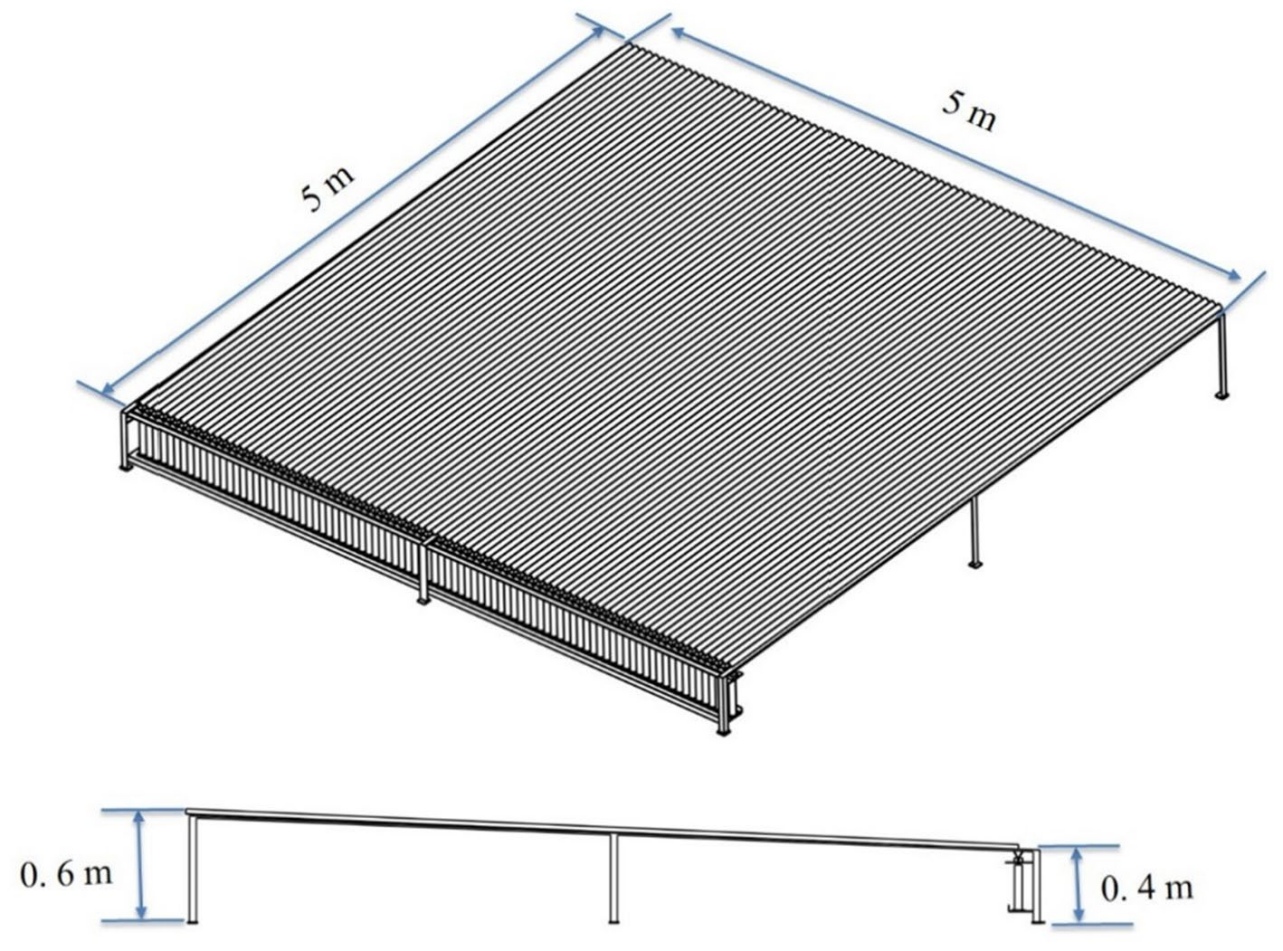
Isometric view of spray patternator.

**Figure 8:**
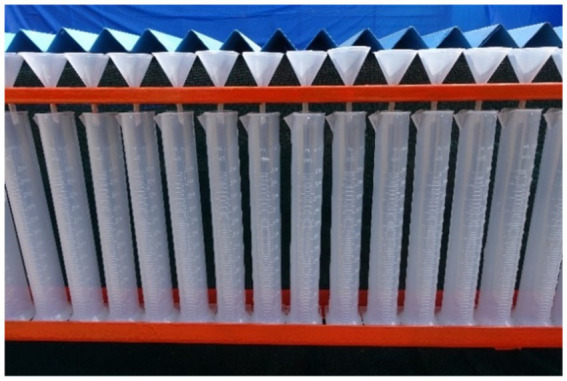
Arrangement of measuring jar in spray patternator.

**Figure 9:**
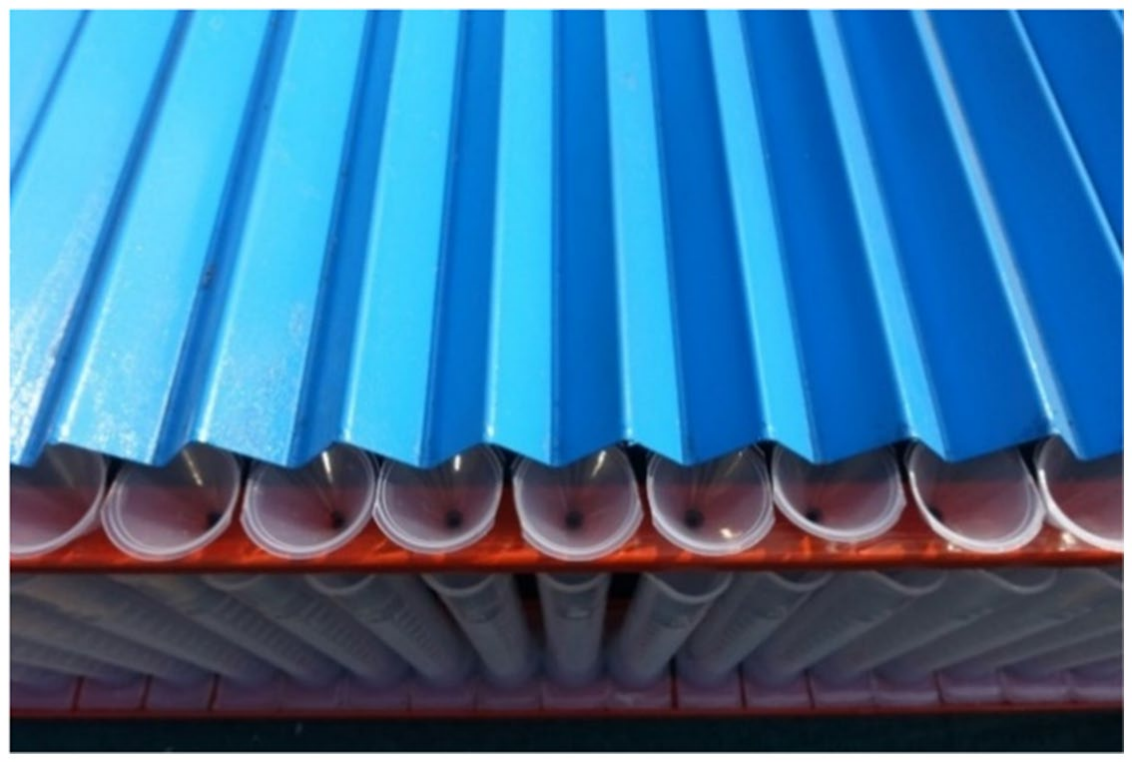
Arrangement of funnel in spray patternator.

**Figure 10:**
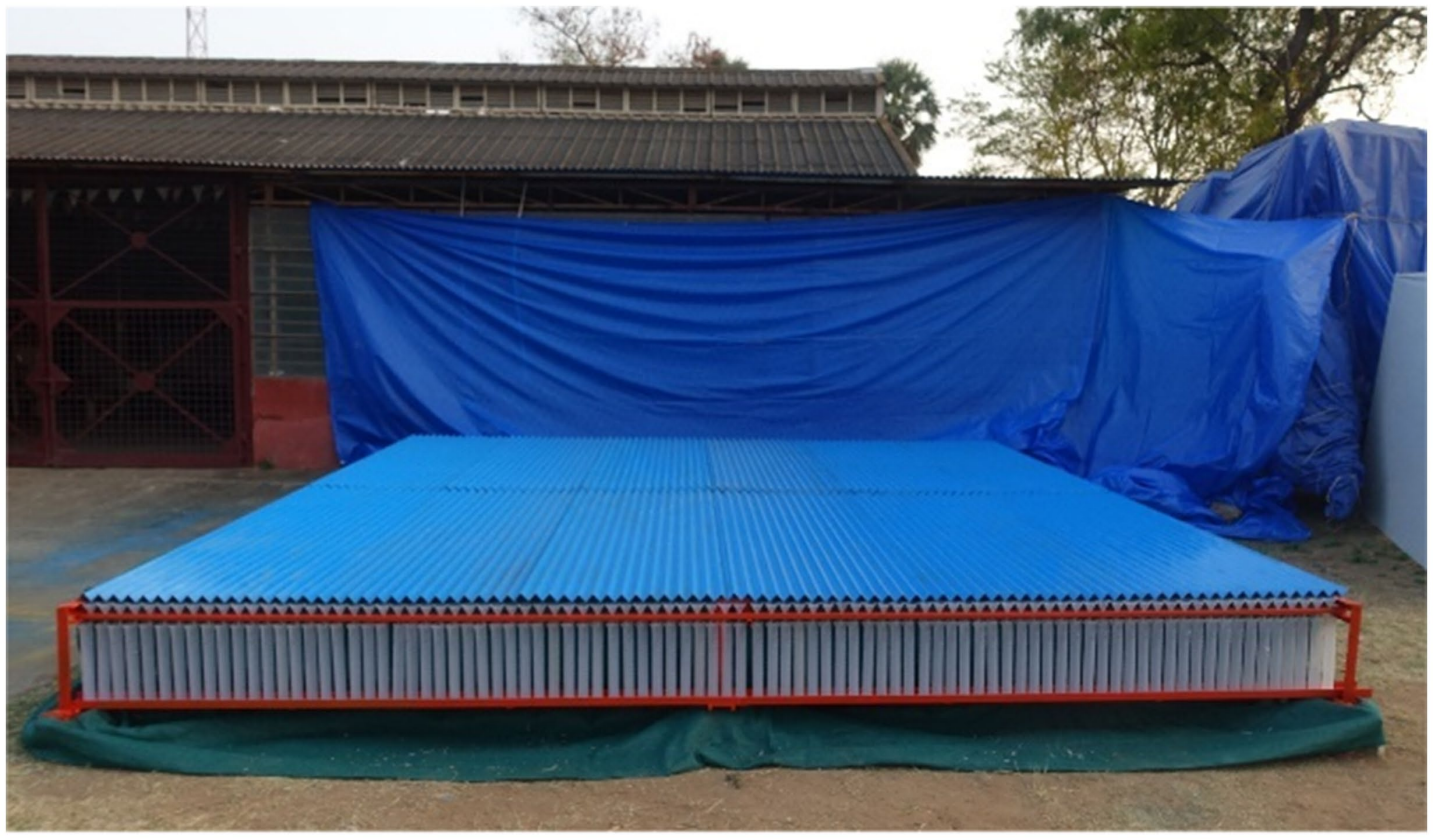
Developed spray patternator for spray volume distribution measurement.

A spray patternator of 5.0 m x 5. 0 m was developed as per the BIS standard (IS: 10064–1982) to study and optimize the spray operational parameters of the drone sprayer *viz.,* height of spray, operating pressure and nozzle mounting configuration under laboratory condition (21). The patternator was fabricated using M.S. channel for the frame and sheet (Figure 7). The spray patternator surface was composed of 0.2 cm thick M.S. sheet positioned horizontally over the frame. The patternator has 91 continuous V- type channels at equal spacing mounted on the rectangular frame. According to IS: 8548 and IS 10064 standards, channels should have 25 ± 0.25 mm width and 100 mm depth (20, 21). These constraints make patternator difficult and costly to develop. Bended M.S. sheet in V shape channels with 55 mm width is more than the recommended width to eliminate splash-back between the measurement grooves due to high downwash airflow produced by the rotor propellers of the drone sprayer. The rectangular frame on which sheets were placed, was made up of 5 mm × 5 mm L-shaped MS channel. Measuring cylinders of 190 mL capacity were placed below each channel to collect the spray liquid. The arrangement of measuring jars and funnel in spray patternator is shown in Figures 8, 9 respectively. Patternator has 25° slope for easy movement of water to the jar. The developed spray patternator is shown in Figure 10. The specifications are mentioned in Table 2.

**Table 2:** Speciation of developed spray patternator.

Main parameter		Norms and numerical value
Overall Size, (L × W × H), mm		5,000 × 5,000 × 600
Support frame structure		L-shaped M.S. channel
Sheet material	Size (L x W), mm	2,500 × 1,250
	Material	M.S sheet
	Number of sheets	12
V channel	Numbers	91
	Width, mm	55
	Depth, mm	35
Patternator inclined slope, degree		25
Number of measuring cylinders		91

### 2.4 Optimization the nozzle spacing and operating pressure for boom and hexa nozzle configuration attachment drone

The experiment was conducted for spray volumetric distribution patterns using a specially designed and fabricated spray patternator at the Agricultural Machinery Research Centre (AMRC), Department of Farm Machinery and Power Engineering, Agricultural Engineering College and Research Institute, Tamil Nadu Agricultural University, Coimbatore (11.0122° N, 76.9354° E). The Drone sprayer volume distribution test was conducted as per the IS: 8548 and IS: 10064 and ASAE (S386.2) standards (20–22).

#### 2.4.1 Experimental setup

**Figure 11:**
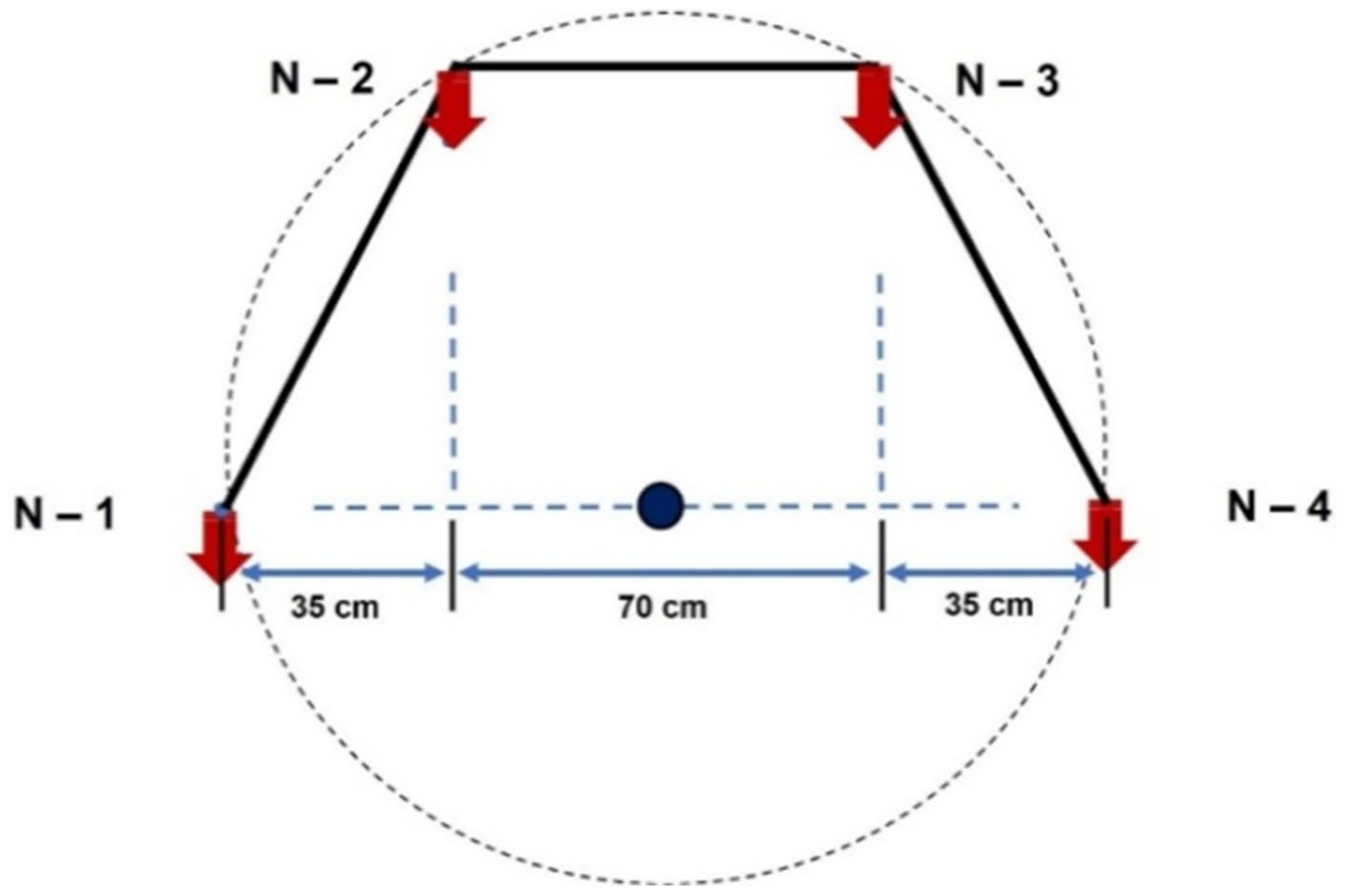
Hexa standard type spray nozzle arrangement.

**Figure 12:**
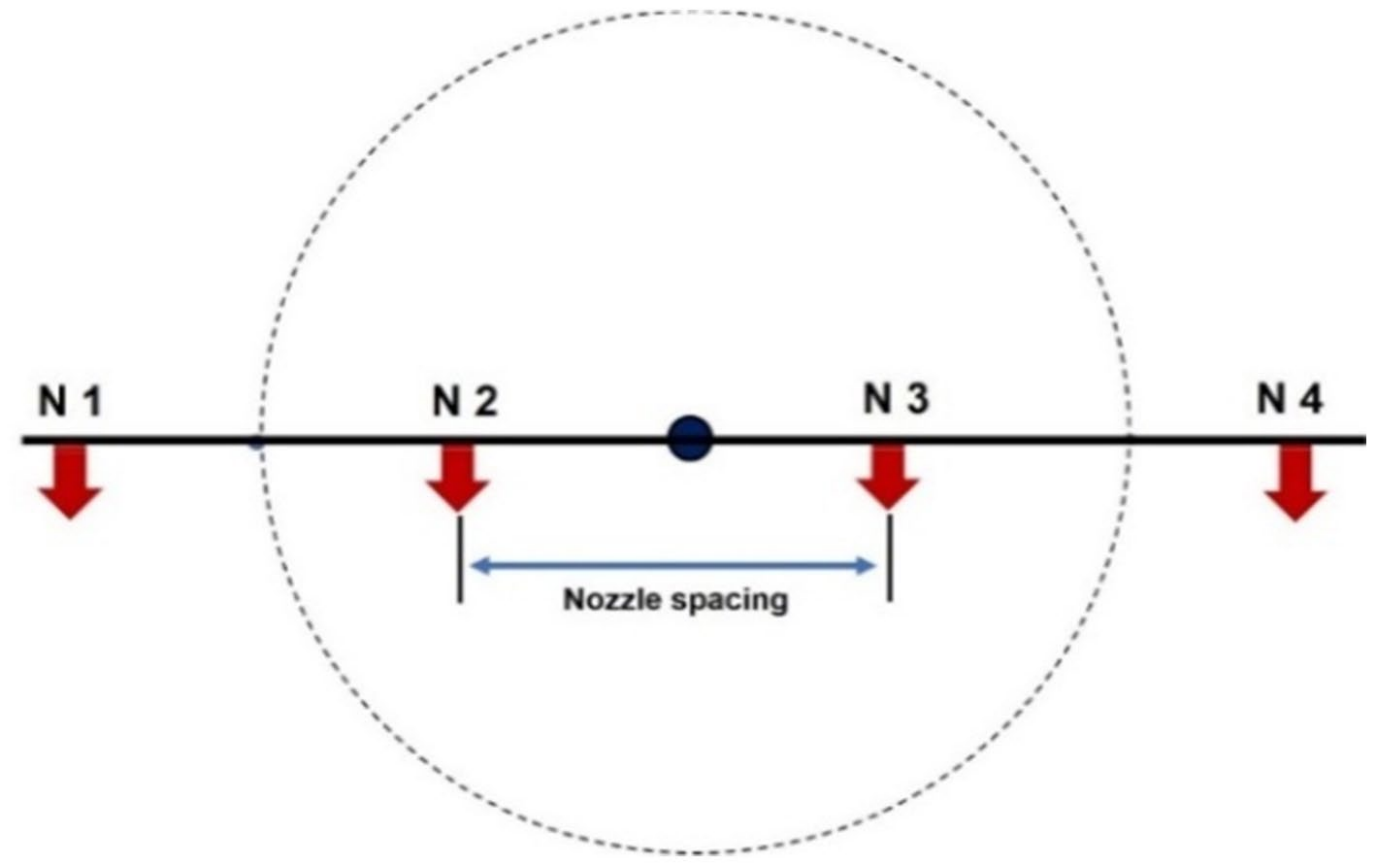
Boom type spray nozzle arrangement.

Two types of nozzles mounting configuration *viz.,* boom and hexa standard type were used in drone sprayer to understand the spray volume distribution system. In the hexa standard type nozzle configuration (Figure 11), four nozzles were mounted below the rotors of the drone sprayer as per the motor BLDC motor configuration and another set of four numbers of flat fan nozzles were placed on the boom type nozzle configuration at equal interval distance (Figure 12).

**Figure 13:**
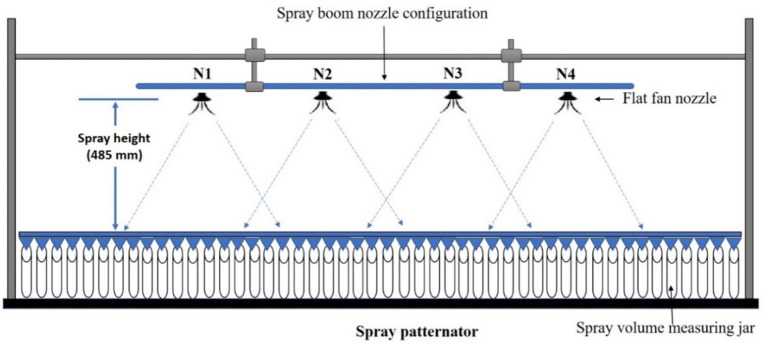
Experimental layout for mounting of spray boom type nozzles arrangement on spray patternator.

**Figure 14:**
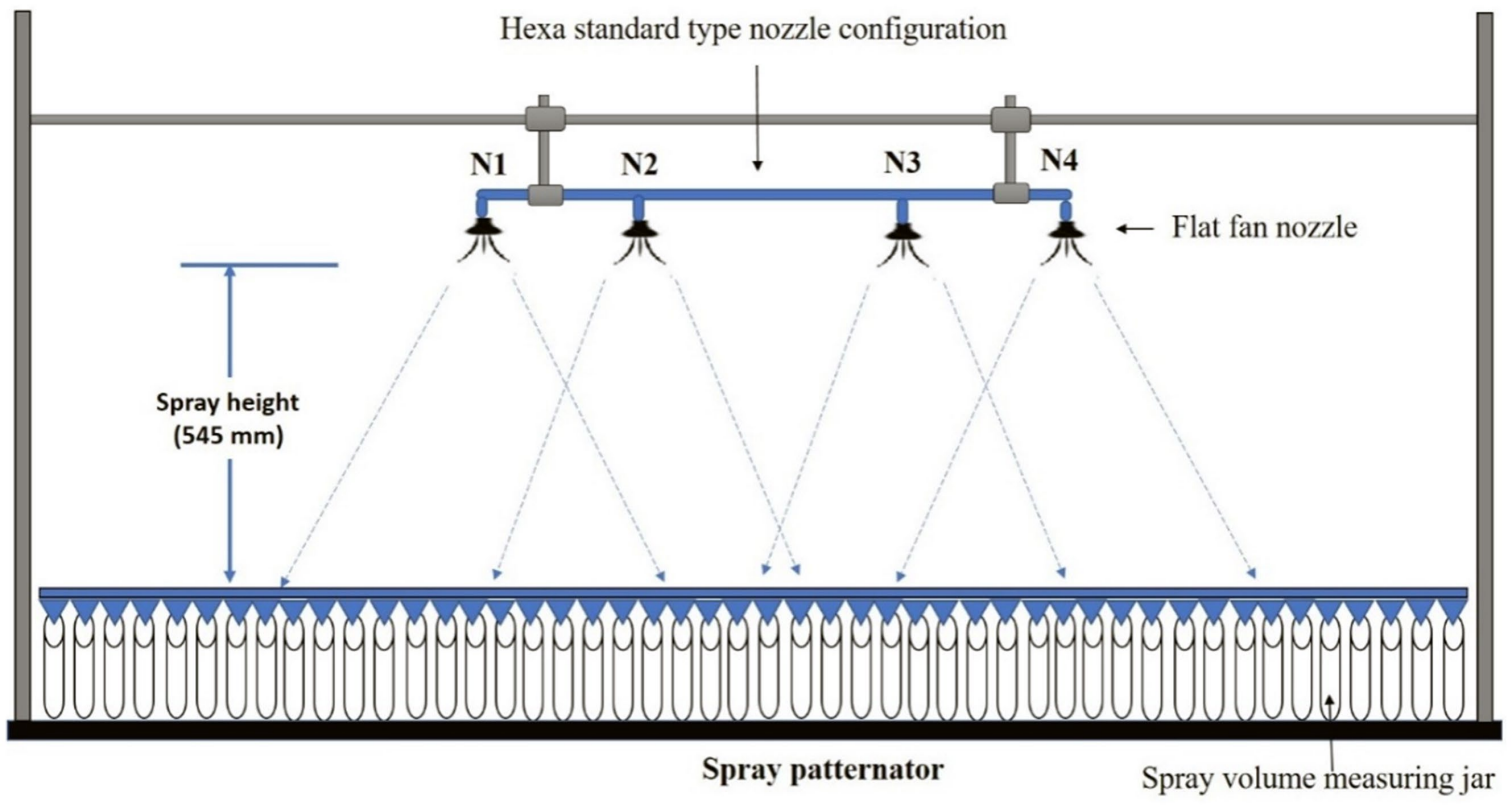
Mounting of nozzle in hexa standard arrangement on spray patternator.

The nozzle spacing of boom type nozzle configuration was optimized at three operating pressures (3.0, 4.0 and 5.0 kg cm^−2^) and three nozzles spacing (0.30, 0.45 and 0.60 m). The height of spray 0.545 m is the distance between the tip of the nozzle to the top of patternator V channel surface was selected as per the recommendation IS: 3652 for flat fan nozzle. A Light Detection and Ranging (LIDAR) distance meter instrument (M/s, DEKOPRO, LRE520 80 M) was used to adjust the height of spray. The spray liquid was horizontally directed and landed on the equidistance V shaped channels. When the fluid reaches the patternator surface, it will be collected in different channels. Each channel is provided with own graduated cylinder at the base of the patternator. The spray liquid in the graduated cylinder of the patternator was collected and the quantity of liquid from each channel was measured and noted. The layout of boom and standard hexa type nozzle configuration with patternator is shown in Figures 13, 14 respectively.

#### 2.4.2 Analysis of spray distribution system

The coefficient of uniformity and spray width were the two main parameters for optimizing the nozzle spacing and operating pressure. These parameters directly influence work efficiency and spray quality.

#### 2.4.3 Liquid distribution uniformity coefficient

The liquid distribution uniformity coefficient (CV) compiles all the patternator data points and summarizes them into a simple percentage, indicating the amount of variation within a given distribution. The uniformity coefficient (CV) (Equations 1–3) is commonly used to quantify the uniformity of spray systems; higher CV values indicate poor uniformity in the spray pattern and the uniformity coefficient is calculated according to the following equation (12, 13):


(1) $$CoefficientofVariation\left(CV\right)=\frac{SD}{X}x\;100\;$$

(2) $$Mean\left(X\right)=\frac{\sum X_{i}}{N}$$

(3) $$Standarddeviation\left(SD\right)=\sqrt{\frac{\sum_{1}^{N}{\left(X_{i}-X\right)}^{2}}{N-1}}$$

Where,

CV – Liquid distribution uniformity coefficient.

X – Volume of liquid contained in specific container, ml.

X_i_ – Average volume of liquid, ml.

N – Number of analysed containers.

Spray distribution uniformity can be obtained with a low coefficient of variation. The above procedure was followed throughout this investigation to determine the coefficient of variation of spray uniformity distribution of the drone sprayer with boom arrangement.

#### 2.4.4 Effective spray width

The effective spray width is the distance between the points on either side of a single swath where the deposit rate equals one-half of the effective application rate. The effective spray width was determined in a manner that will give the most uniform overall application rate.

### 2.5 Test of spray volume distribution pattern in hover outdoor condition

Uniformity coefficient was selected to study and optimized nozzle spacing and operating pressure of operational parameters boom and standard hexa nozzle configuration attachment to drone sprayer in outdoor condition.

#### 2.5.1 Experimental setup

**Figure 15:**
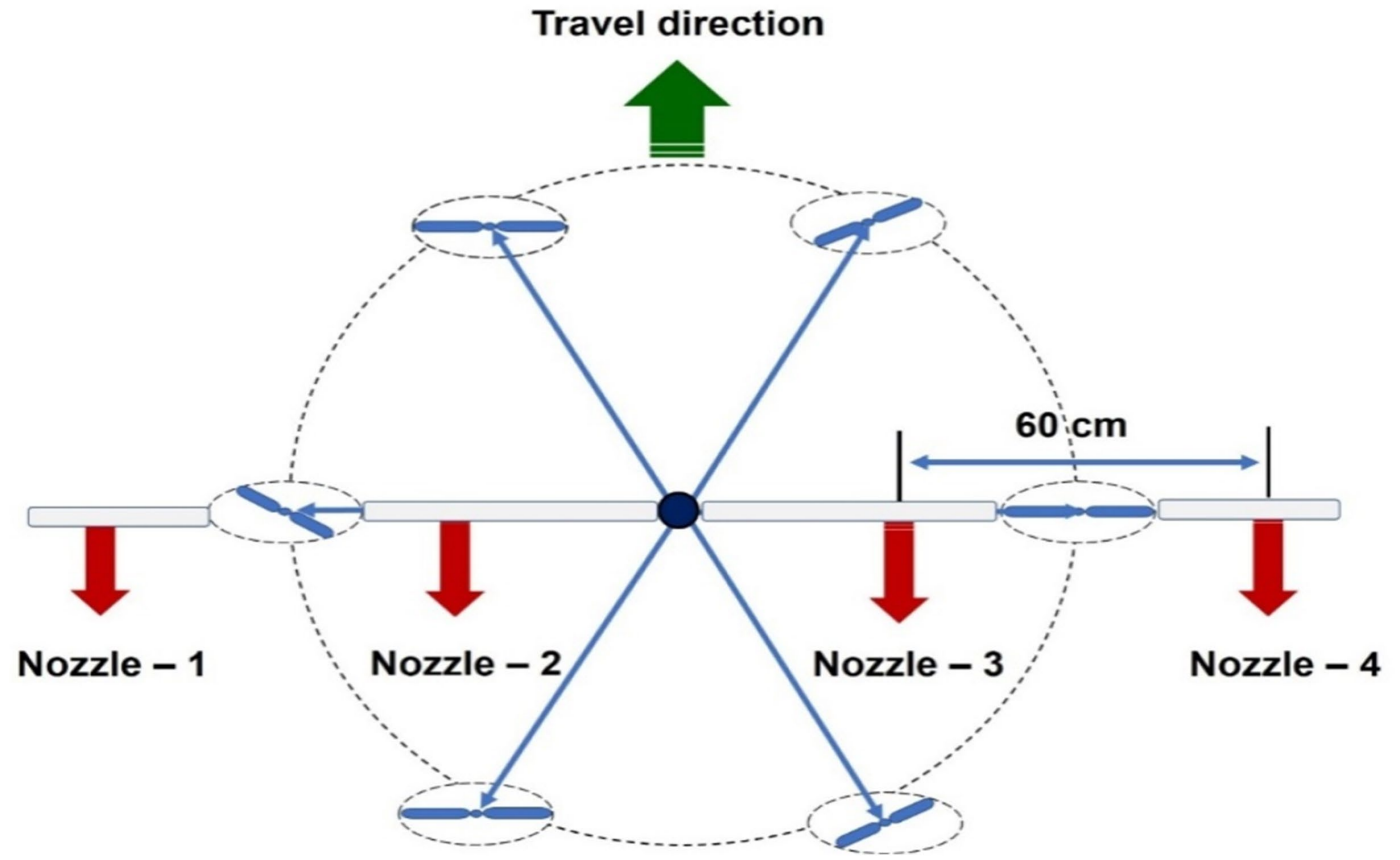
Top view of mounting of spray boom type nozzle arrangement to drone sprayer.

**Figure 16:**
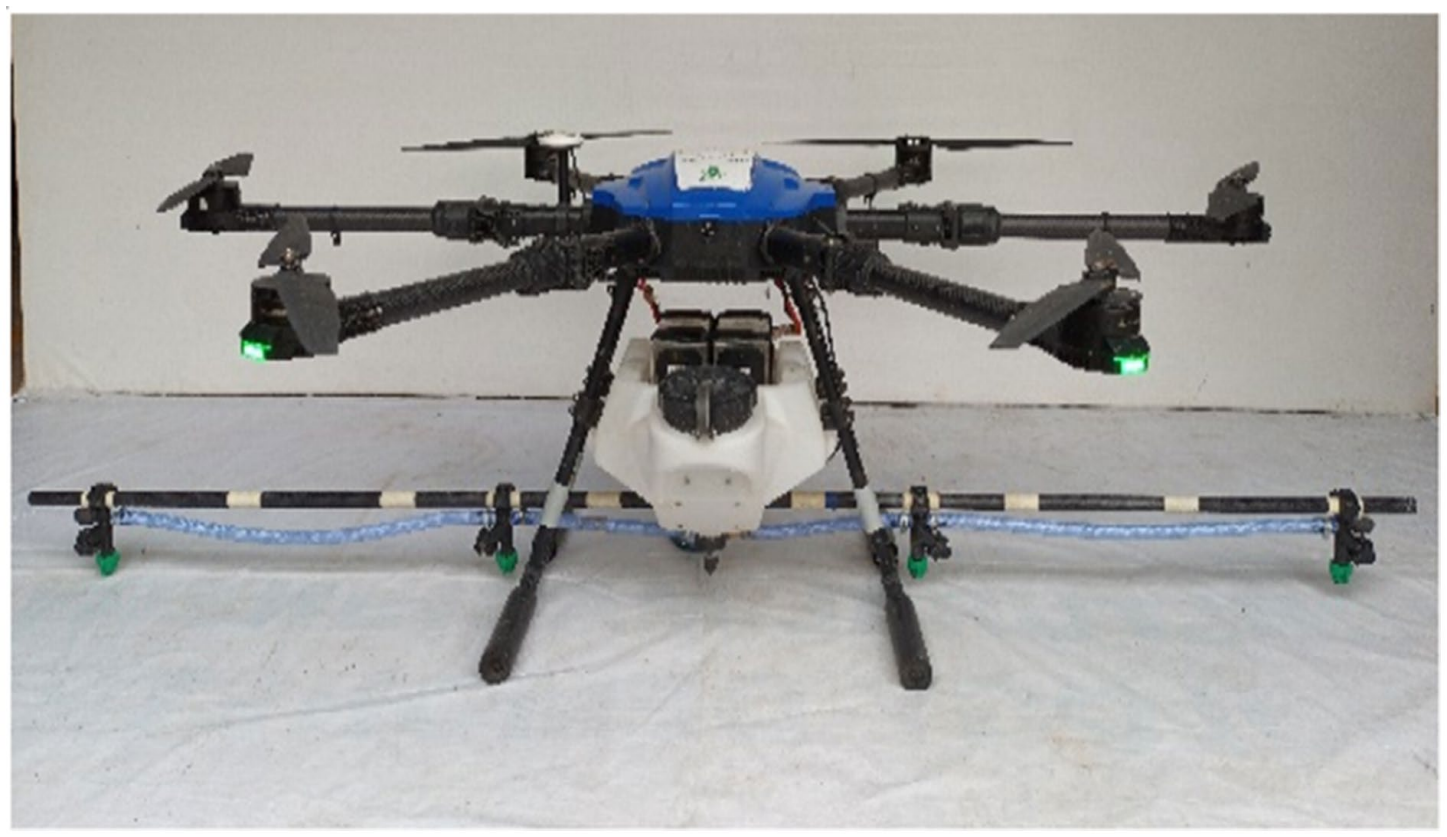
Arrangement of spray nozzles on boom configuration and attachment to Drone sprayer.

**Figure 17:**
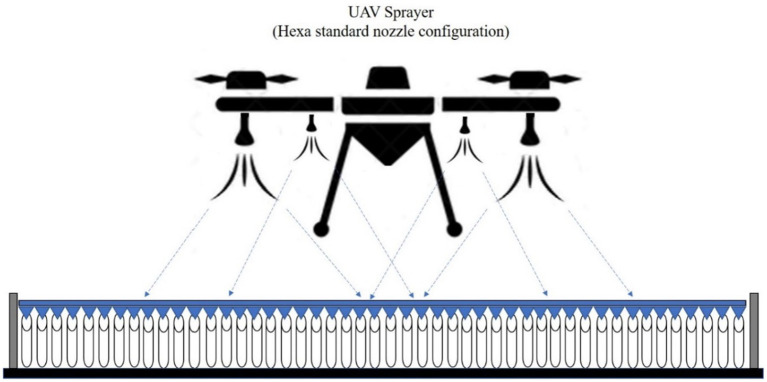
Schematic diagram of nozzles arrangement in hexa standard configuration attachment to drone sprayer for outdoor test.

**Figure 18:**
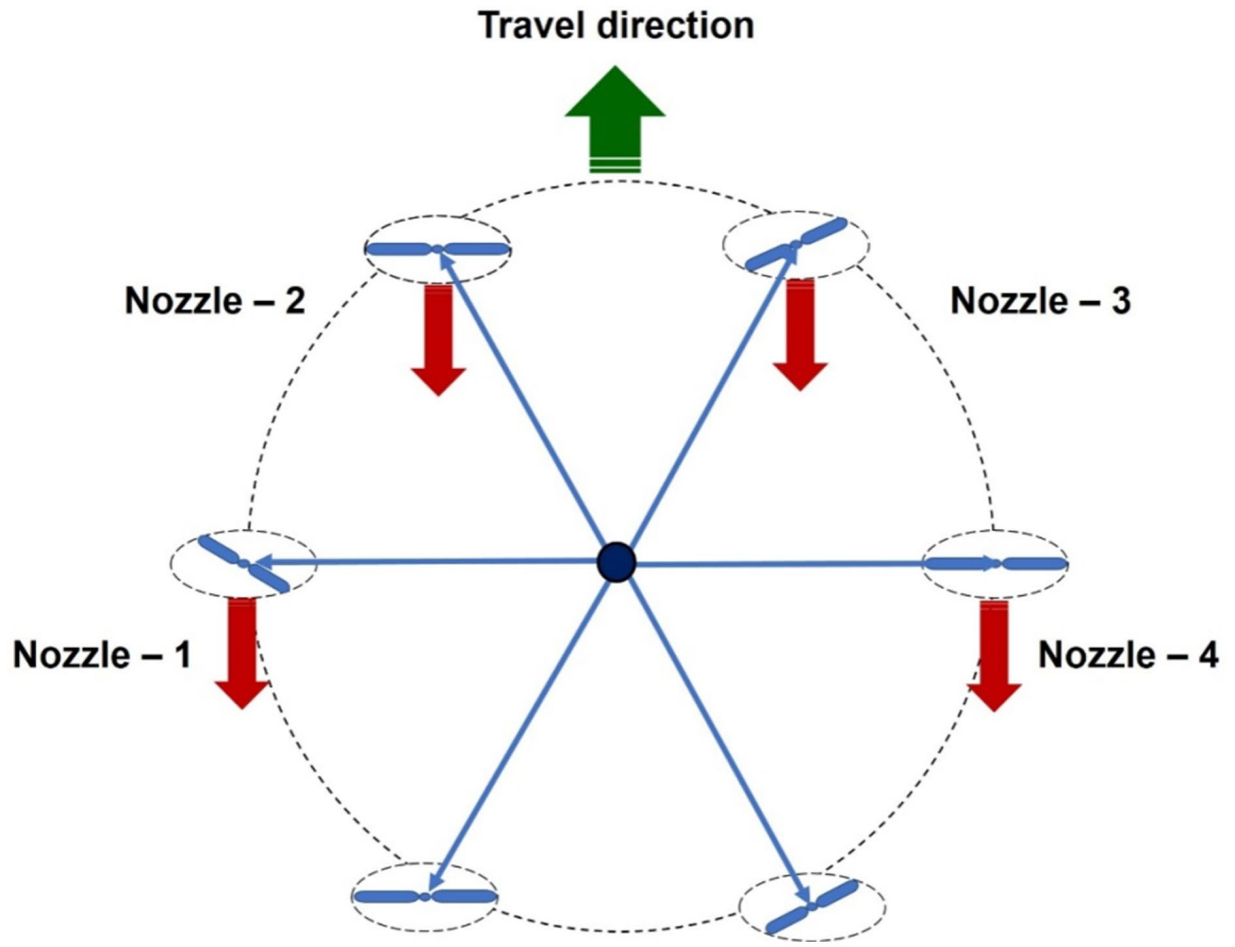
Top view of mounting of hexa standard arrangement type nozzle arrangement to drone sprayer.

Four flat fan nozzles were mounted on the boom with optimized nozzle spacing (0.60 m) and attached below the drone sprayer fluid tank and landing gear structure. The arrangement of optimized nozzle spacing on the spray boom and attachment to drone sprayer is shown in Figures 15, 16. Another set of four nozzles were mounted below the BLDC rotors as a hexa standard nozzle configuration attachment and is shown in Figures 17, 18.

**Figure 19:**
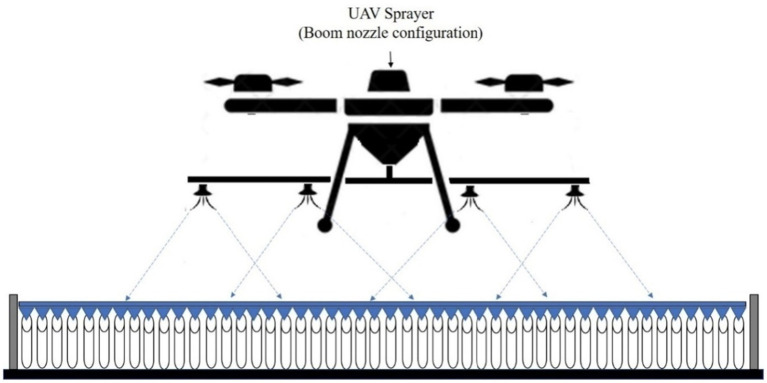
Schematic diagram of nozzles arrangement in boom configuration attachment to drone sprayer for outdoor test.

To record and analyse the spray volume distribution pattern for boom and hexa standard nozzle configuration, the drone sprayer hovered at three flight heights *viz.,* 1.0 m, 2.0 m, and 3.0 m. These are the independent variables that mainly influence the functional performance of the drone spray volume distribution pattern in terms of quantity of spray volume collected (ml), coefficient of uniformity (%) and spray width (mm). For each treatment, a 10 litre water tank was filled and the spray volume was measured in each measuring jar. Each treatment was carried out three times. The coefficient of uniformity and spray width were calculated for three spray hover heights. This spray volume distribution pattern test procedure was followed as per IS: 8548 and ASAE (S386.2) standards (20, 22). Figure 19 and Supplementary Figures S1, S2 show the volumetric distribution of the drone sprayer with boom and hexa standard nozzle configuration in the patternator and the volume of liquid collected in the measuring jar.

### 2.6 Statistical analysis

A study included two independent variables: (1) nozzle spacing (i.e., 300, 450 and 600 mm), (2) operating pressure (i.e., 3, 4 and 5 kg cm^−2^), and two dependent variables (i.e., uniformity distribution and spray width) with three replications. The factorial CRD design for the analysis of variance (Two-way ANOVA) tests to determine if there are significant differences between the spraying nozzle spacing and operating pressure. All tests were replicated thrice and the statistical analysis was carried out in OPSTAT Software (O. P. Sheoran, a computer programmer at CCS HAU, Hisar, India) to determine the level of significance.

### 2.7 Recording of meteorological parameters during outdoor condition test

During the drone spray volume distribution pattern test for optimizing the height of spray and nozzle configuration, the different meteorological parameters *viz.,* air temperature, wind velocity, humidity and rainfall were recorded during at outdoor condition. A portable anemometer was mounted on a square iron pipe (20 x 20 x 2 mm) at 2.0 m above the ground level to measure the wind velocity. Weather conditions, including wind speed, air temperature, and relative humidity during the study, are presented in Table 3.

**Table 3:** Meteorological data during the spray volume distribution pattern test.

Environmental parameters	Values
Air temperature, °C	28.3 to 30.9
Relative humidity, %	54.5 to 60.2
Wind velocity, ms^−1^	0.11 to 0.21
Rainfall, mm	0

## 3 Results and discussion

### 3.1 Results of drone spray operational parameters

The spray operational parameters such as operating pressure, nozzle discharge rate and spray angle were measured using standard procedure. The mean value of total spray discharge rate, spray angle and operating pressure of combined four nozzles at different motor speed mode of 0, 10, 20, 30, 40, 50, 60, 70, 80, 90, and 100% of PWM is furnished in Table 4.

**Table 4:** Results of spray motor speed, pressure, nozzle flow rate and nozzle spray angle.

Motor speed mode (%)	Motor speed (RPM)	Pressure (kg cm^−2^)	Discharge rate (lm^−1^)	Spray angle (degree)
10	247.0	0.2	1.0	66.52
20	606.0	0.3	1.5	71.25
30	1192.3	0.6	1.8	75.92
40	1730.3	1.5	2.1	86.87
50	2197.7	3.0	2.8	92.67
60	2781.0	3.7	3.0	97.07
70	3394.7	4.0	3.2	99.32
80	4017.3	4.3	3.3	101.25
90	4491.0	4.6	3.3	103.37
100	4514.0	5.0	3.4	105.17

From Table 4, it was observed that the spray discharge rate and spray angle increased with the increase in spray operating pressure. Spray angle was measured using digital protractor and shown in Supplementary Figure S3. Among the 10 different modes of spray motor speed, 50, 70 and 100% modes were selected as it produced discharge rate 2.8 L m^−1^, 3.1 L m^−1^and 3.4 lm^−1^ at 3 kg cm^−2^, 4 kg cm^−2^and 5 kg cm^−2^, respectively for the investigation on the spray volume distribution pattern of boom and hexa standard nozzles experiment in hover condition.

### 3.2 Results of spray volume distribution pattern for single nozzle

The volume discharge rate of single nozzle was tested at a different pressure level of 3.0, 4.0 and 5.0 kg cm^−2^ on the patternator in the laboratory prior to the outdoor test trials. Spray pattern at 4.0 kg cm^−2^ pressure was found to have uniform distribution pattern based on the CV. The spray pattern of a single flat fan nozzle at different operating pressures are shown in Supplementary Figure S4.

The volume of liquid collected from each container in respect to the total volume of liquid collected from all the containers and the coefficient of variation was calculated for different pressures. The standard bell curve was observed at all the nozzle pressures. Based on coefficient of variation (53.0%), the spray volume distribution pattern was found to be high at 4.0 kg cm^−2^ nozzle pressure. From the laboratory test, an optimized nozzle pressure of 4.0 kg cm^−2^ was maintained to test the drone spray with different nozzle arrangements at outdoor condition.

### 3.3 Effect of nozzle spacing and operating pressure on spray uniformity

The effect of nozzle configuration, operating pressure and nozzle spacing on spray uniformity distribution and spray width was analyzed. The spray distribution pattern test for optimization of nozzle spacing and operating pressure for both boom type and hexa standard nozzle configuration based on coefficient of variation is presented in Table 5 and Supplementary Figure S5. The minimum coefficient of variation (CV) represents the better spray uniformity distribution Luck et al. (12) and Padhee et al. (13).

**Table 5:** Results on effect of type of nozzle configuration, nozzle spacing and operating pressure on uniformity of distribution and spray width.

Type of Nozzle configuration	Nozzle spacing (mm)	Operating pressure (kg cm^−2^)	Uniformity of distribution, CV (%)	Spray width (mm)
Boom	300	3	52.08	2034
		4	55.80	2,145
		5	54.29	2,145
	450	3	45.72	2,530
		4	44.61	2,640
		5	44.21	2,640
	600	3	37.63	3,025
		4	36.99	3,235
		5	38.35	3,235
Hexa	35/70	3	49.36	2,530
		4	48.31	2,695
		5	50.57	2,751

From Table 5 and Supplementary Figure S5, for boom spray arrangement, the maximum coefficient variance (CV) value was found to be 52.08% at 3 kg cm^−2^ operating pressure and 300 mm nozzle spacing. The minimum CV value was found to be 36.99% at pressure of 4.0 kg cm^−2^ and 600 mm nozzle spacing. Luck et al. (12) and Padhee et al. (13) presented spray liquid uniformity distribution was better with lower CV value. Hence on the spray uniformity distribution results in Table 5, the optimized nozzle spacing of 600 mm and a 4.0 kg cm^−2^ operating pressure were selected with lower CV value 36.99% for boom spray arrangement on the drone sprayer for hover condition test. The maximum spray width value was found to be 3,235 mm at 4 kg cm^−2^ operating pressure and 600 mm nozzle spacing. The minimum spray width value was found to be 2,035 mm at pressure of 3.0 kg cm^−2^ and 300 mm nozzle spacing.

In the hexa standard type nozzle configuration, four nozzles were mounted below the rotors of the drone sprayer. The spray distribution pattern test for optimization of operating pressure for hexa standard nozzle configuration based on coefficient of variation is presented in Table 5. From Table 5 and Supplementary Figure S6, for hexa standard nozzle arrangement, the maximum CV value was found to be 50.57% at 5 kg cm^−2^ operating pressure. The minimum CV value was found to be 48.31% at pressure of 4.0 kg cm^−2^. Based on the spray uniformity distribution value, the optimized 4.0 kg cm^−2^ operating pressure was selected for hexa standard spray arrangement on the drone sprayer for hover condition test.

Supplementary Table S1 presents the statistical analysis, i.e., analysis of variance showing the *p*-value for dependent variable at 5% significance level. A p-value above 0.05 was observed for uniformity of distribution and spray width. Therefore, the nozzle spacing had a significant effect on uniformity distribution and spray width. Operating pressure shows not significant in uniformity distribution and significant in spray width. There is significant interaction effect between nozzle spacing and operating pressure for spray width.

### 3.4 Spray volume distribution pattern of drone sprayer in hover at outdoor condition

The spray volume distribution pattern test was conducted and analyzed to optimize the height of spray based on coefficient of variation at outdoor condition. For boom type nozzle configuration, four numbers of flat fan nozzles were placed with 0.60 m spacing and in the hexa standard nozzle configuration, four nozzles were mounted below the rotors of the drone sprayer operated at 4.0 kg cm^−2^ operating pressure. The drone sprayer with boom spray nozzle configuration hovered at three heights of spray *viz.*, 1.0, 2.0 and 3.0 m and spray volume were collected from each jar during outdoor conditions (average wind speed, air temperature and relative humidity were measured as 0.19 m s^−1^, 28.3°C and 57%, respectively). The effects of nozzle configuration and hover height on spray uniformity distribution, spray width and total quantity of liquid collected were analyzed in single pass distribution method and presented in Supplementary Table S2.

#### 3.4.1 Effect of nozzle configuration and height of spray on spray uniformity

According to the coefficient of variation results presented in Supplementary Table S2, the spray height has a significant impact on spray uniformity distribution. Lower spray uniformity distribution (57.21%) was found at 1.0 m height of spray for hexa standard nozzle configuration. Similarly, the maximum spray uniformity distribution was found as 47.26% at 2.0 m height of spray. It was also observed that when the drone sprayer hover height was increased from 1.0 m to 2.0 m from the top of the patternator, better spray uniformity distribution was recorded. When the height was increased from 2.0 m to 3.0 m from the top of the patternator, the volume of liquid collected and uniformity of distribution from the target area was reduced, which may be due to side drift. In boom nozzle configuration, lower spray uniformity distribution of 58.42% was found at 1.0 m hover height. Similarly, the maximum spray uniformity distribution of 54.80% was observed at 2.0 m hover height. It was also observed that when the drone sprayer hover height was increased from 1.0 m to 2.0 m from the top of the patternator, better spray uniformity distribution was found for both nozzle configuration.

#### 3.4.2 Effect of nozzle configuration and height of spray on spray width

From Supplementary Table S2, the spray width of hexa standard nozzle arrangement was found to be minimum, (3,145 mm) for 1.0 m hover height, whereas it was found to be maximum, (3,865 mm) at 2.0 m hover height. In boom nozzle configuration, the minimum spray width was found as 4,450 mm for boom nozzle arrangement at 1.0 m height, which was higher (3,145 mm) than the hexa standard nozzle arrangement at the same hover height. The maximum spray width was found as 4,902 mm at 2.0 m height of boom spray. It was observed that the spray width increased by increasing the height of spray from 1.0 to 2.0 m from the patternator. The height of spray did not influence the discharge rate during the laboratory trials. Generally, it was observed that the spray width of boom nozzle arrangement is higher than the hexa standard nozzle arrangement.

#### 3.4.3 Effect of hover height on quantity of liquid collected

In hexa standard nozzle configuration, four numbers flat fan nozzles *viz.*, N1, N2, N3 and N4 are mounted below the rotor propeller. The horizontal distance between the nozzles N1 to N2 and N3 to N4 were measured as 35 cm, whereas the distance between the nozzles N2 to N3 was measured as 70 cm. The spray volume was collected at three hover heights, *viz.*, 1,000, 2,000 and 3,000 mm from the top of the patternator surface and results are presented in Supplementary Table S1 and Supplementary Figure S7. When compared to the height of spray, the volume of water collected at the central portion of the drone sprayer was less (5,389 mL) when hovered at 1.0 m height compared to 2.0 m (5,949 mL) and 3.0 m (5,559 mL) heights.

From the Supplementary Table S2 and Supplementary Figure S8 for boom nozzle configuration, it was found that, due to downwash air flow and increase in horizontal distance between the nozzles N2 and N3, the volume of liquid collected below the drone was less, irrespective of the height of operation of the drone above the patternator, it was also observed that more round vertex patterns were generated at 1.0 m hover height compared to the hover height of 2.0 and 3.0 m due to the direct impact of downwash airflow generated by the rotor propellers on the droplets. At 1.0 m hover height, most of the spray droplets were distributed back to the upper side and did not move towards the downside V-channel surface of the patternator. The liquid collected at the center of drone sprayer was less (5,190 mL) at 1.0 m hover height, whereas it was 6,230 mL and 5,146 mL at 2.0 m and 3.0 m hover heights, respectively.

Supplementary Table S3 presents the statistical analysis, i.e., analysis of variance showing the *p*-value for dependent variable at 5% significance level. Therefore, the nozzle configuration, height of spray had a significant effect on uniformity distribution, spray width and quantity of liquid collected. There is significant interaction effect between nozzle configuration and height of spray for uniformity of distribution, spray width and quantity of liquid collected.

### 3.5 Spray volume distribution pattern test in one direction application method

In one direction application method, the overlaps between two passes were considered. The spray uniformity and effective spray width were calculated and the results are presented in Supplementary Table S4.

According to the coefficient of variation results in Supplementary Table S4 and Supplementary Figures S9, S10, spray height has a significant impact on spray uniformity distribution. Lower spray uniformity distribution with maximum CV was found as 19.83 and 21.62% and at 1.0 m height of spray for boom and hexa standard nozzle configuration. Similarly, the maximum spray uniformity distribution with minimum CV was found as 18.90 and 17.95% at 2.0 m height of spray boom and hexa standard nozzle configuration. It was also observed that when the drone sprayer hover height was increased from 1.0 m to 2.0 m from the top of the patternator, better spray uniformity distribution was found.

Similarly, the minimum spray width was found to be 2,805 mm and 2,035 mm at 1.0 m height of spray for boom and hexa standard nozzle arrangement, respectively. The maximum spray width was found to be 3,190 mm and 2,310 mm at 2.0 m height of spray for boom and hexa standard nozzle arrangement, respectively. It was observed that the spray width was increased by increasing the height of spray from 1.0 m to 2.0 m from the patternator. The effective spray width in one direction spray distribution for boom nozzle configuration at 1.0, 2.0 and 3.0 m height of spray is shown in Supplementary Figure S10. Similarly, for hexa standard nozzle configuration, the spray distribution pattern is given in Supplementary Figure S9.

Supplementary Table S5 presents the statistical analysis, i.e., analysis of variance showing the *p*-value for dependent variable at 5% significance level. A *p*-value above 0.05 was observed for uniformity of distribution for nozzle configuration. Therefore, the height of spray had a significant effect on uniformity distribution and spray width. There is significant interaction effect between nozzle configuration and height of spray for uniformity of distribution and spray width.

In boom nozzle arrangement (Supplementary Figure S9), it was also observed that more round vertex patterns were generated during the 1.0 m and 3.0 m hover height compared to the 2.0 m hover height due to the direct impact of downwash airflow generated by the rotors. At 1.0 m hover height, most of the spray droplets were distributed back to the upper side and did not move towards the downside V-channel surface of the patternator. In hexa standard nozzle arrangement (Supplementary Figure S10), it was also observed that there was less round vertex pattern generated during hexa configuration nozzle spray compared to boom type nozzles due to the direct impact of downwash airflow generated by the rotor propeller. The downwash airflow produced by the rotor propellers reduced the liquid distribution uniformity coefficient and significantly influenced the change of the lateral distribution pattern of spray drops produced by the flat fan spray nozzles. Similarly, as in previous research works Berner and Chojnacki (14) and Qing et al. (15) there was a change in the shape of liquid deposition on the patternator due to the influence of downwash airflow produced by the drone rotor propellers.

Similarly, as in previous research works Yallappa et al. (11) and Pachuta et al. (16) the asymmetry of the airflow distribution generated by the drone rotors with respect to the nozzle axis is what causes the lateral spray liquid distribution of the settled liquid on the patternator to change shape. The volume of the liquid that was deposited in the patternator later grooves also varied significantly Chojnacki and Pachuta (17). A higher spray distribution amount of the liquid was sprayed from the twin flat nozzle than from the single flat nozzle (18). Earlier reported work was done at a constant spray height, where in the present investigation, the results were obtained at varying sprays heights (1.0, 2.0 and 3.0 m) and nozzle spacing (30, 45, 60 cm) at an optimized operating pressure (5.0 kg cm^−2^). The results showed that there were obvious differences in the distribution of spray volume patterns for boom and hexa configuration nozzles.

## 4 Conclusion

Food and nutritional security is the situation where in public around the globe, in all conditions must maintain constant physical and financial, ensuring reliable global access to sufficient nutritious and safe food. Results of in this experiment are study and optimized the spray operational parameters *viz.*, height of spray, nozzle spacing and spray operating pressure. Nozzle spacing and operating pressure for boom and hexa standard nozzle configuration to drone sprayer was optimized by using developed spray patternator (5.0×5.0 meter). The optimized nozzle spacing of 0.6 m and a 4.0 kg cm^−2^ operating pressure was chosen for the drone sprayer distribution test at outdoor conditions based on the spray uniformity distribution value. The spray volume distribution for both boom and hexa standard nozzle arrangement in hover condition, observed that when the drone sprayer hover height was increased from 1.0 m to 2.0 m from the top of the patternator, better spray uniformity distribution, spray width and quantity of liquid collected was recorded. The central portion of the patternator collected less water (5,194 mL) when a drone sprayer hovered at 1.0 m height compared to 2.0 (5,416 mL) and 3.0 m (6,231 mL) hover heights. With the increase of hover height, the change of the downwash airflow led to a gradual decrease in spray volume distribution in the effective spray area. A better spray uniformity distribution was found when the drone sprayer hover height was increased from the top of the patternator. A more round spray droplet vertex pattern was generated during the 1.0 m hover height compared to the 2.0 and 3.0 m hover heights due to the direct impact of downwash airflow generated by the rotors. Downwash airflow produced by rotor propellers reduced the liquid distribution uniformity coefficient and significantly influenced the change of lateral distribution pattern of spray drops produced by the flat fan spray nozzles. Thus, the drone sprayer should be operated at an appropriate spray height of 2.0 m to attain the recommended application rate of pesticides. The good spray uniform distribution was found in hexa configuration nozzle arrangement as compared to the boom arrangement of nozzles. The study provides references for the height of spray and different nozzle configuration arrangement to drone sprayer for efficient operation. Lastly, the study demonstrates that optimizing drone sprayer parameters, such as spray height and nozzle configuration, ensures efficient pesticide application, leading to enhanced food and nutritional security by promoting sustainable agricultural practices under varying climate conditions.

## Data Availability

The datasets presented in this study can be found in online repositories. The names of the repository/repositories and accession number(s) can be found in the article/Supplementary material.
