# Supplementary material for: Improving agricultural spraying with multi-rotor drones: a technical study on operational parameter optimization
Source: Front Nutr. 2024 Dec 18;11:1487074. doi: 10.3389/fnut.2024.1487074 (PMC11688191; doi:10.3389/fnut.2024.1487074)
Supplement: Supplementary file 1 [file Table_1.pdf]

Supplementary Tables

Supplementary Table 1: The variance analysis showing the p value for dependent variable at 5% significance level for nozzle spacing and operating pressure.

**Parameters**	**Uniformity of distribution, CV (%)**	**Spray width (mm)**
Nozzle spacing	0.00	0.0
Operating pressure	0.67	0.0
Nozzle spacing x Operating pressure	0.07	0.0

Supplementary Table 2: Spray volume distribution pattern test – single pass distribution pattern

**Type of Nozzle configuration**	**Height of Spray, mm**	**Uniformity of distribution (CV), %**	**Spray width (mm)**	**Quantity of liquid collected (ml)**
**Boom**	1000	58.42	4450	5194
	2000	54.80	4902	6231
	3000	55.20	4750	5416
**Hexa**	1000	57.21	3145	5389
	2000	47.26	3865	5949
	3000	52.86	3303	5559

Supplementary Table 3. The variance analysis showing the p value for dependent variable at 5% significance level for nozzle spacing and height of spray for Single pass distribution pattern

**Parameters**	**Uniformity of distribution, CV (%)**	**Spray width (mm)**	**Quantity of liquid collected (ml)**
Nozzle configuration	0.000	0.000	0.000
Height of spray	0.000	0.000	0.000
Nozzle configuration x Height of spray	0.000	0.000	0.000

Supplementary Table 4: Effect of nozzle configuration and height of spray on uniformity distribution and effective spray width in one-direction application distribution pattern

Type of Nozzle configuration	Height of Spray, mm	One-direction application distribution pattern	
		Uniformity of distribution (CV), %	Effective spray width (mm)
Boom	1000	19.83	2805
	2000	18.90	3190
	3000	19.09	2970
Hexa	1000	21.62	2035
	2000	17.80	2310
	3000	18.36	2210

Supplementary Table 5. The variance analysis showing the p value for dependent variable at 5% significance level for nozzle spacing and height of spray for One-direction application distribution pattern

Parameters	Uniformity of distribution (CV), %	Effective spray width (mm)
Nozzle configuration	0.89	0.0
Height of spray	0.0	0.0
Nozzle configuration x Height of spray	0.0	0.0
