# Supplementary material for: Improving agricultural spraying with multi-rotor drones: a technical study on operational parameter optimization
Source: Front Nutr. 2024 Dec 18;11:1487074. doi: 10.3389/fnut.2024.1487074 (PMC11688191; doi:10.3389/fnut.2024.1487074)
Supplement: Supplementary file 2 [file Image_1.pdf]

Supplementary Images

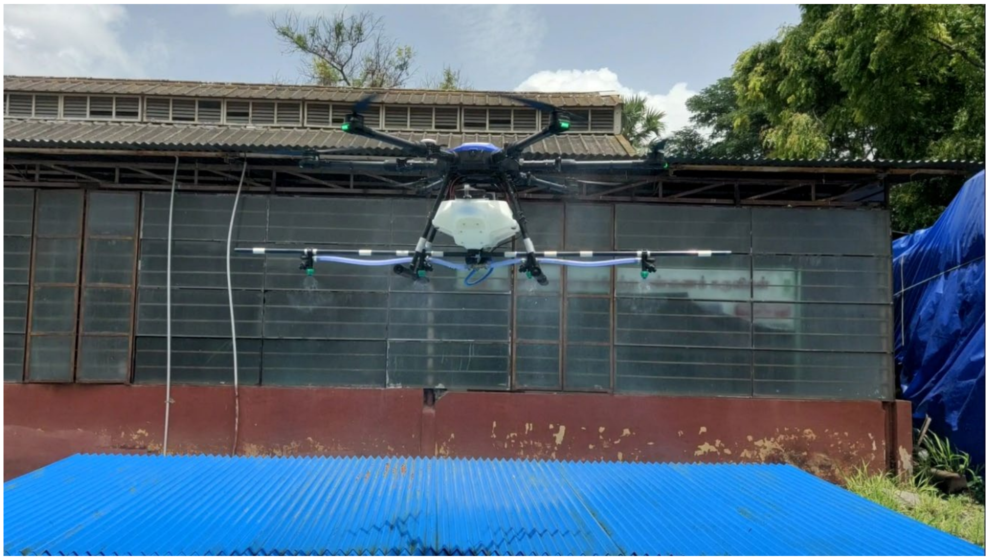

Supplementary Image 1: Drone sprayer with boom spray nozzle configuration for volume distribution test in spray patternator at outdoor conditions

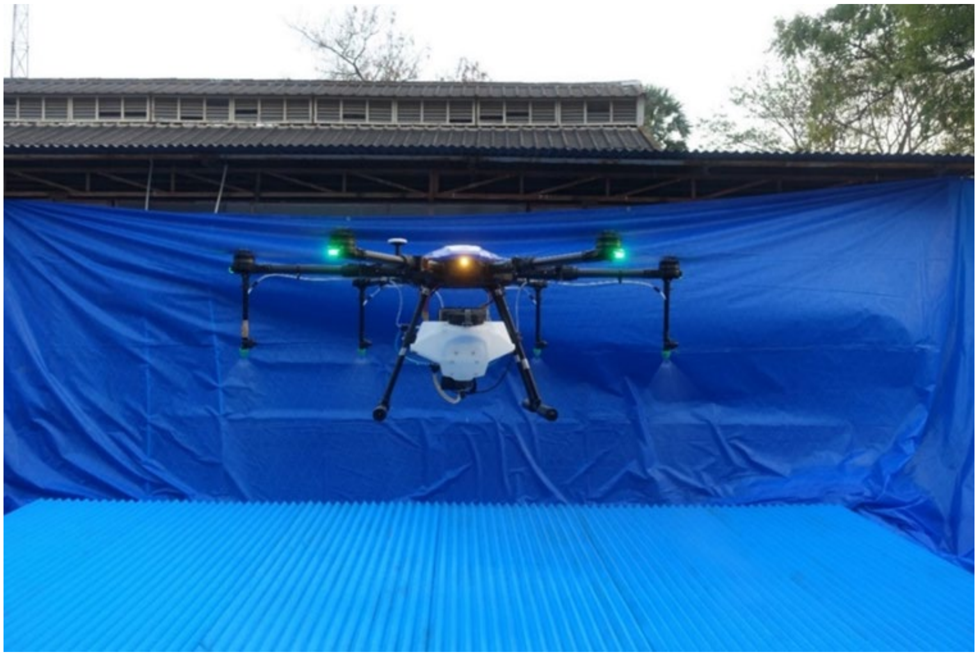

Supplementary Image 2: Drone Sprayer with standard hexa nozzle arrangement for volume distribution test in spray patternator at outdoor condition

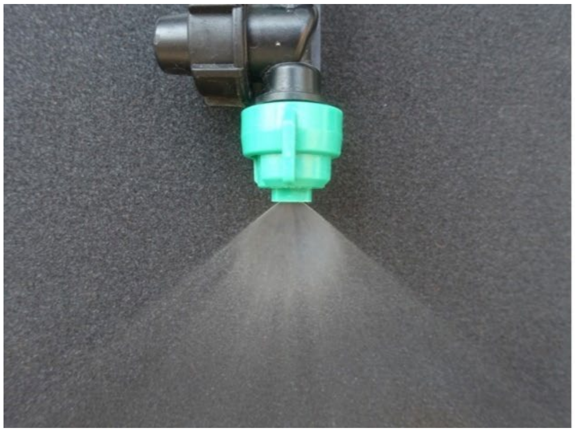

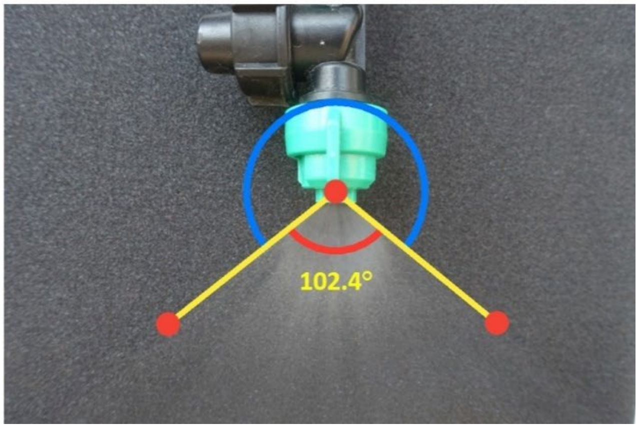

A B

Supplementary Image 3: Spray angle measurement using digital protractor

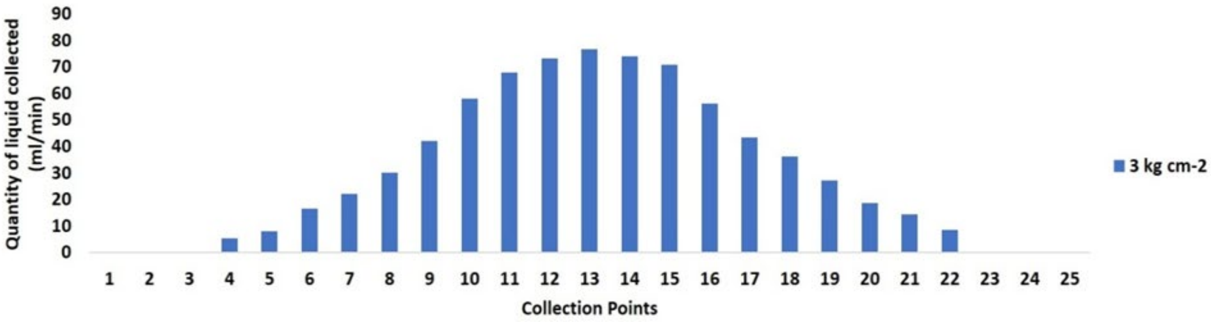

A

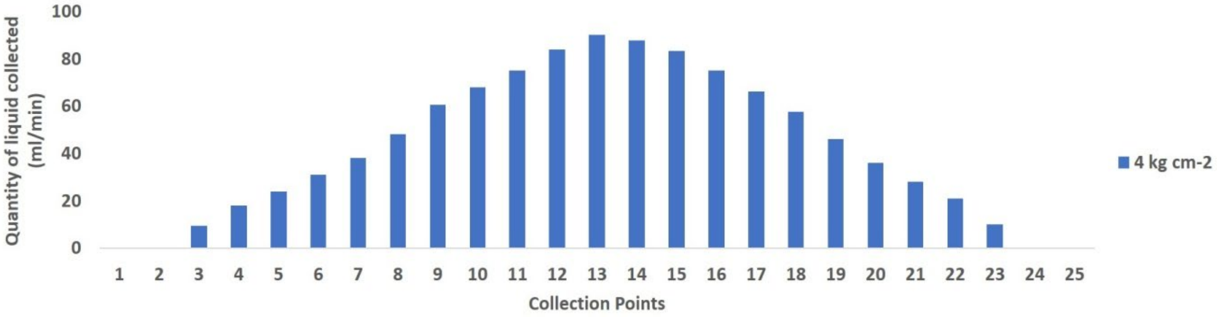

B

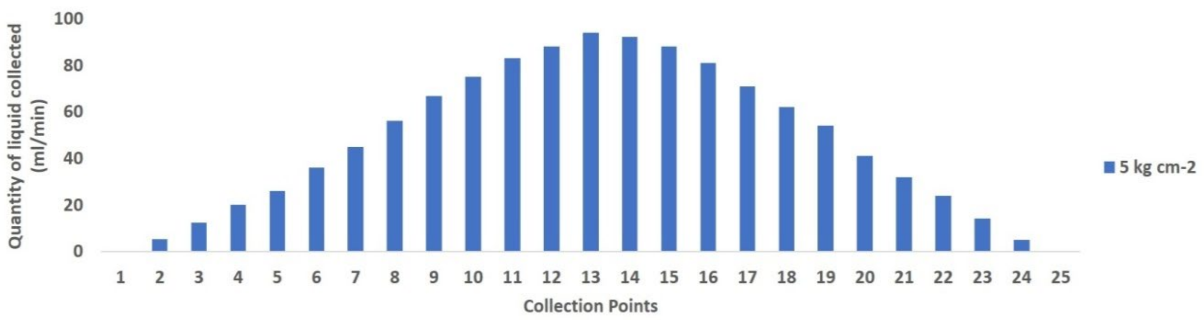

C

Supplementary Image 4: Spray volume distribution pattern of single nozzle at 3.0, 4.0 and 5.0 kg cm^{-2} operating pressure levels on the patternator

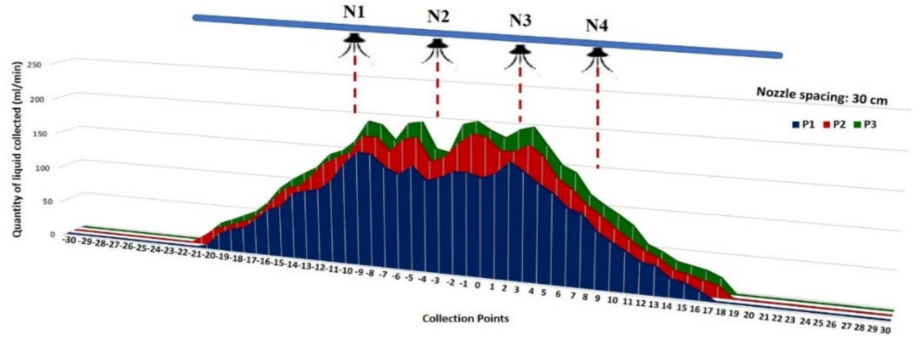

A

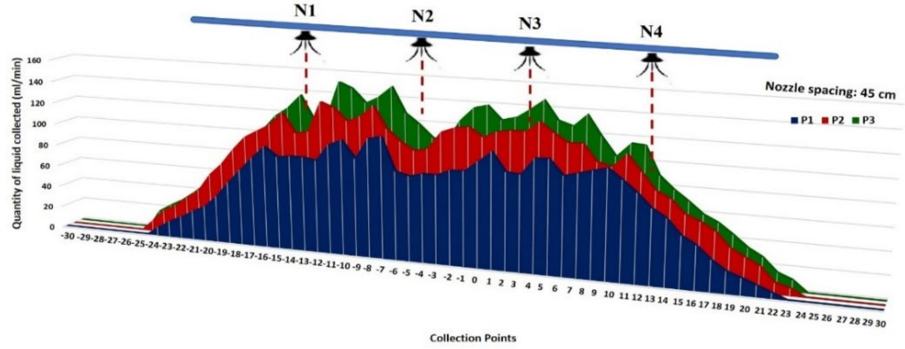

B

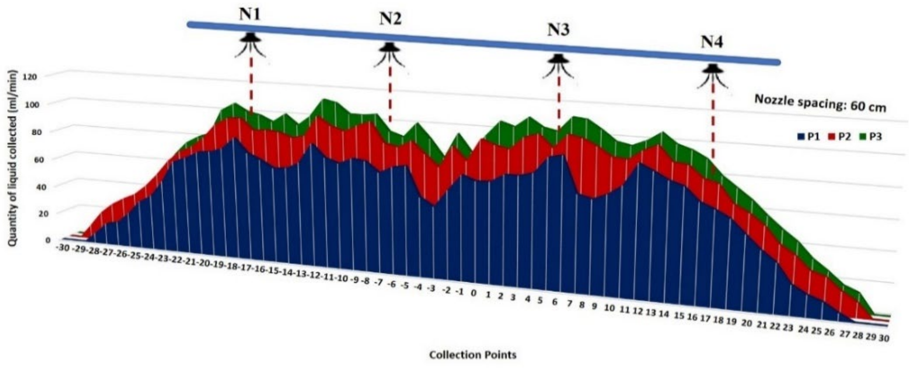

C

Supplementary Image 5: Effect of nozzle spacing and operating pressure on spray volumetric distribution for boom type nozzles configuration: a) 30 cm, b) 45 cm and c) 60 cm nozzle spacing

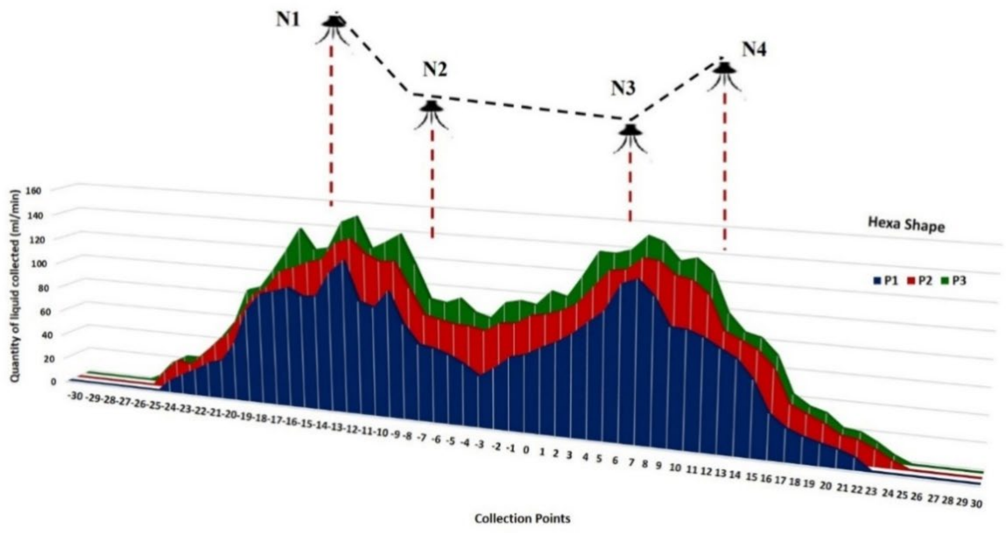

Supplementary Image 6: Spray volume distribution pattern of standard hexa type nozzles configuration

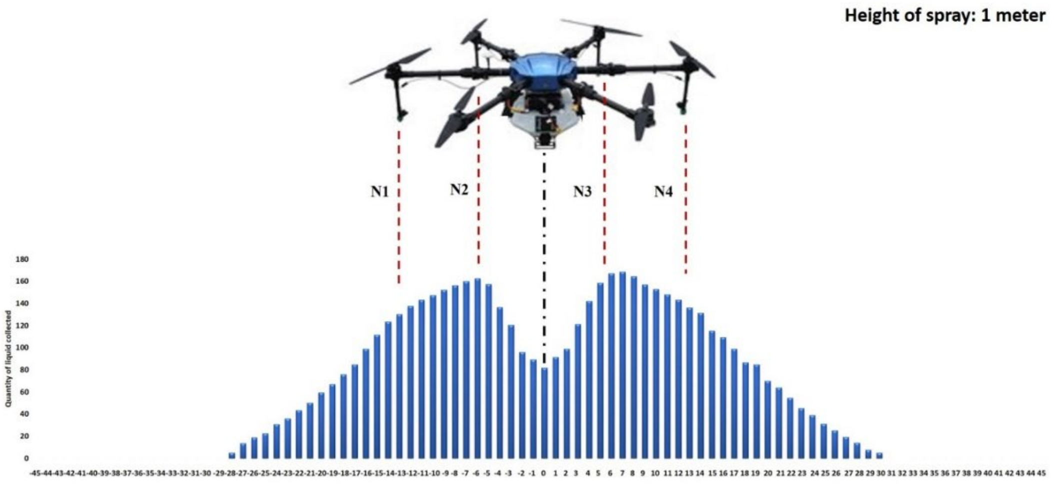

Height of spray: 1 meter
N1
N2
N3
N4
Quantity of liquid collected
0 20 40 60 80 100 120 140 160 180
-45 -44 -43 -42 -41 -40 -39 -38 -37 -36 -35 -34 -33 -32 -31 -30 -29 -28 -27 -26 -25 -24 -23 -22 -21 -20 -19 -18 -17 -16 -15 -14 -13 -12 -11 -10 -9 -8 -7 -6 -5 -4 -3 -2 -1 0 1 2 3 4 5 6 7 8 9 10 11 12 13 14 15 16 17 18 19 20 21 22 23 24 25 26 27 28 29 30 31 32 33 34 35 36 37 38 39 40 41 42 43 44 45

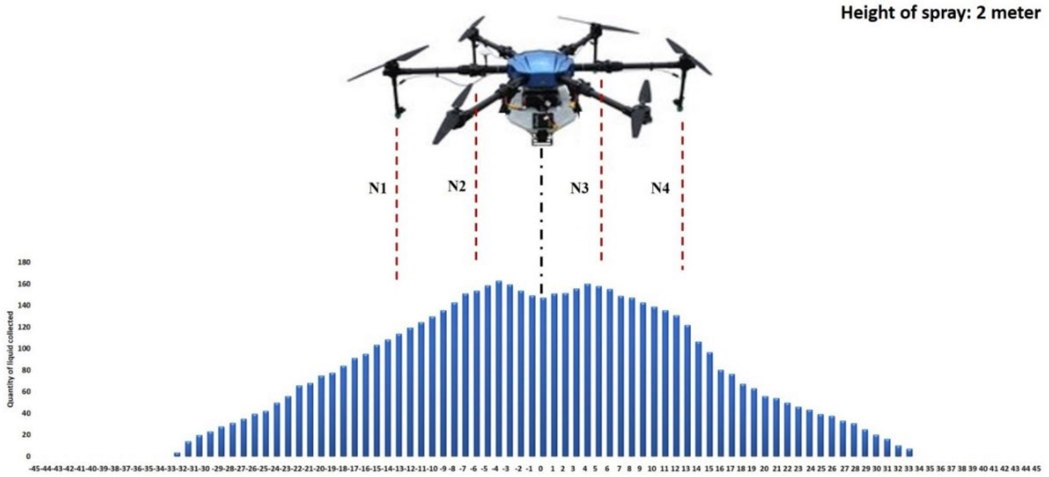

Height of spray: 2 meter
N1
N2
N3
N4
Quantity of liquid collected
0 20 40 60 80 100 120 140 160 180
-45 -44 -43 -42 -41 -40 -39 -38 -37 -36 -35 -34 -33 -32 -31 -30 -29 -28 -27 -26 -25 -24 -23 -22 -21 -20 -19 -18 -17 -16 -15 -14 -13 -12 -11 -10 -9 -8 -7 -6 -5 -4 -3 -2 -1 0 1 2 3 4 5 6 7 8 9 10 11 12 13 14 15 16 17 18 19 20 21 22 23 24 25 26 27 28 29 30 31 32 33 34 35 36 37 38 39 40 41 42 43 44 45

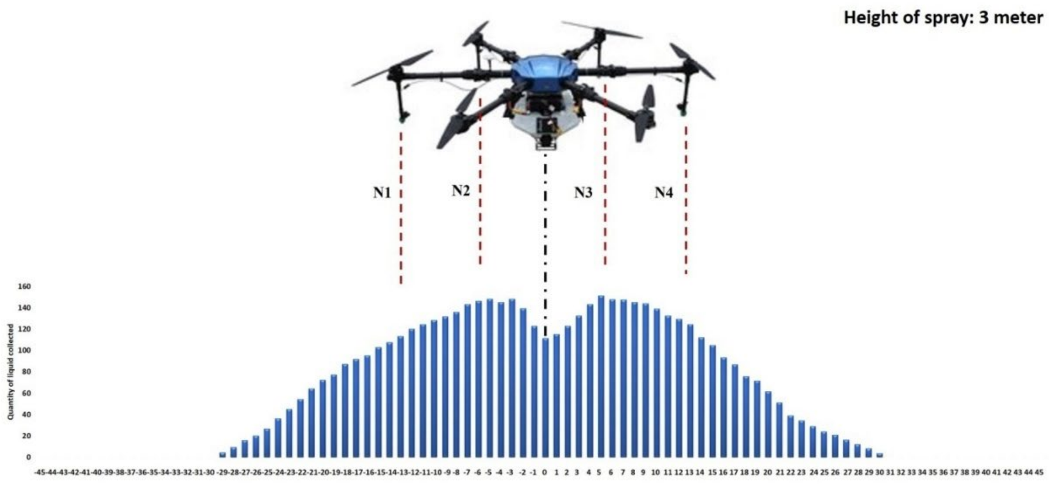

Height of spray: 3 meter
N1
N2
N3
N4
Quantity of liquid collected
0 20 40 60 80 100 120 140 160
-45 -44 -43 -42 -41 -40 -39 -38 -37 -36 -35 -34 -33 -32 -31 -30 -29 -28 -27 -26 -25 -24 -23 -22 -21 -20 -19 -18 -17 -16 -15 -14 -13 -12 -11 -10 -9 -8 -7 -6 -5 -4 -3 -2 -1 0 1 2 3 4 5 6 7 8 9 10 11 12 13 14 15 16 17 18 19 20 21 22 23 24 25 26 27 28 29 30 31 32 33 34 35 36 37 38 39 40 41 42 43 44 45

Supplementary Image 7: Spray volume distribution pattern of standard hexa type nozzles configuration

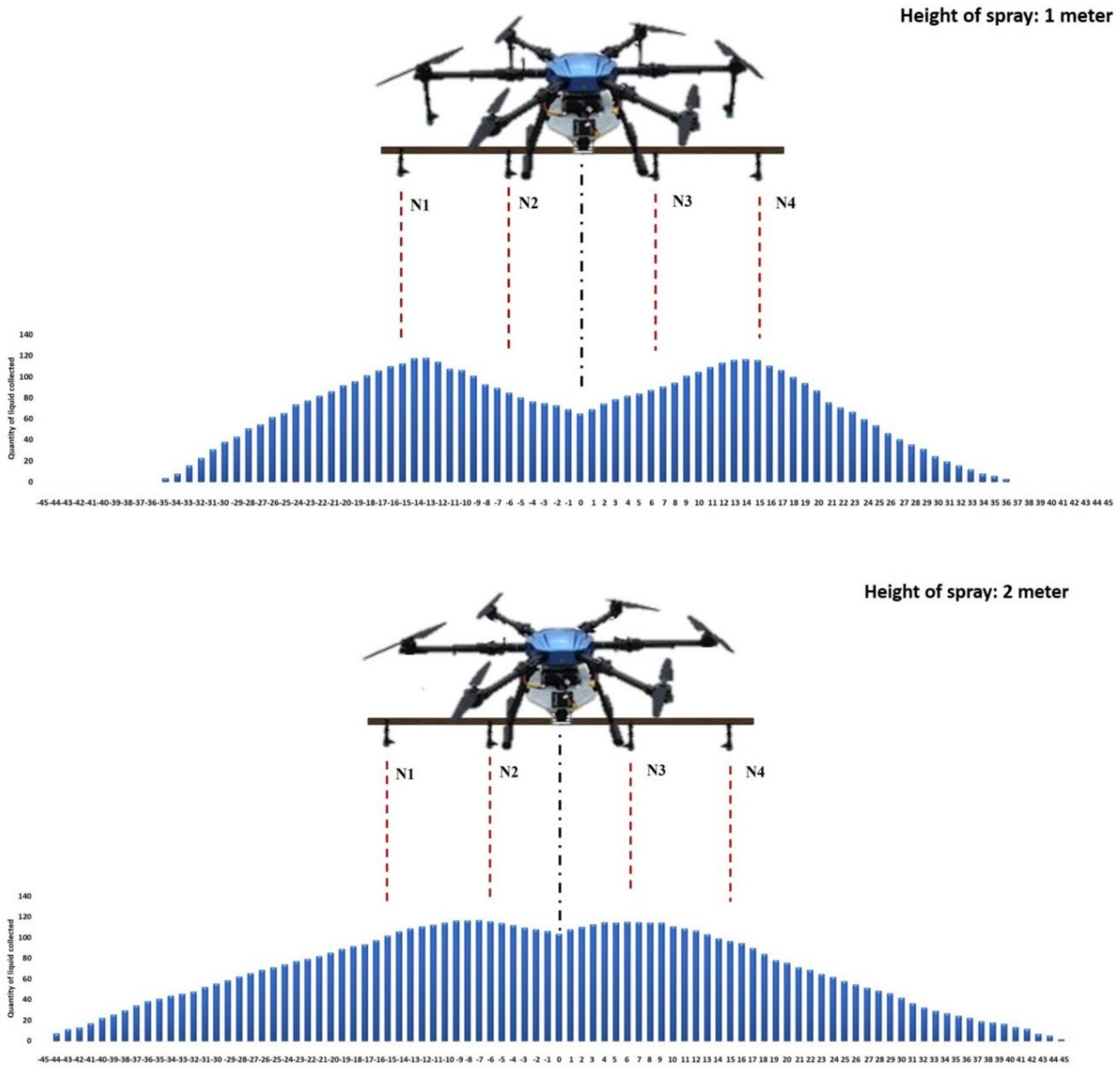

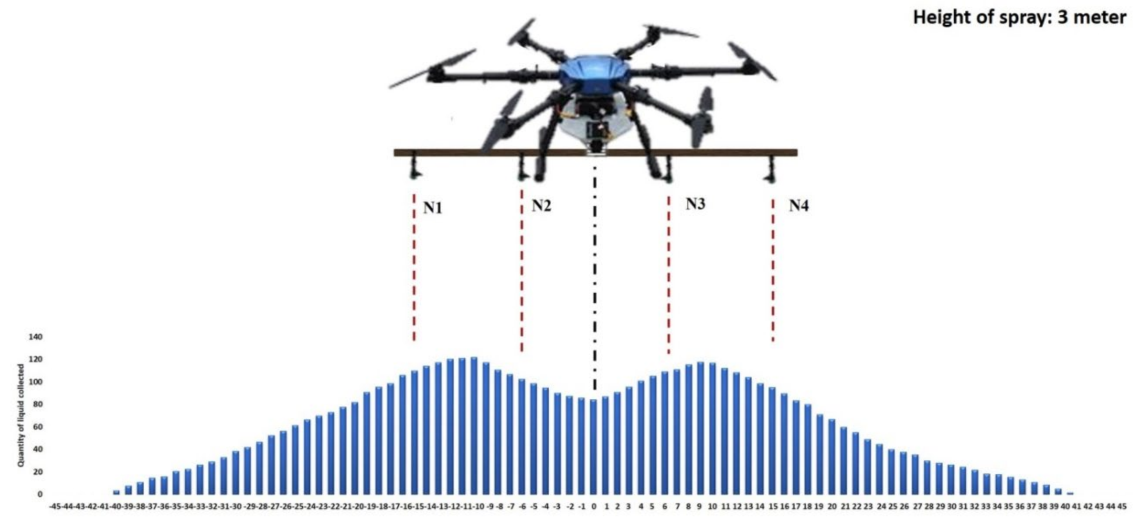

Supplementary Image 8: Spray volume distribution pattern of boom type nozzles configuration

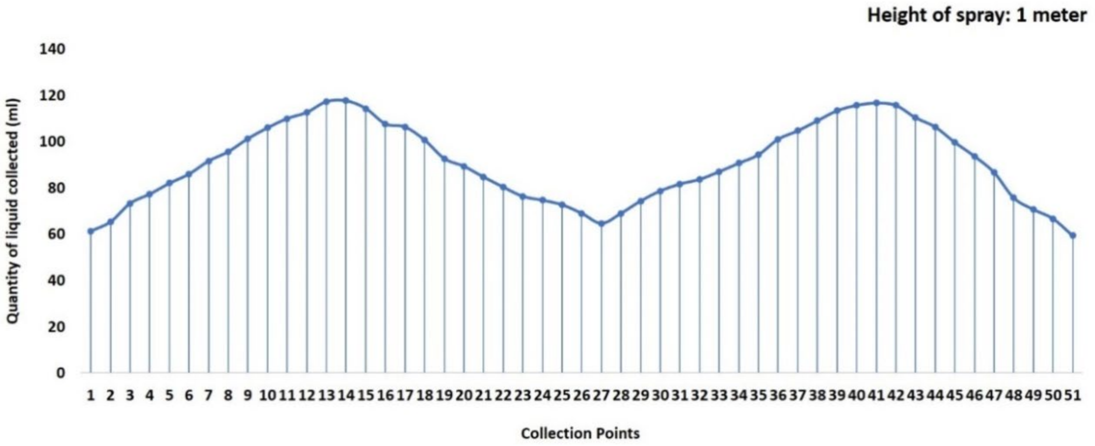

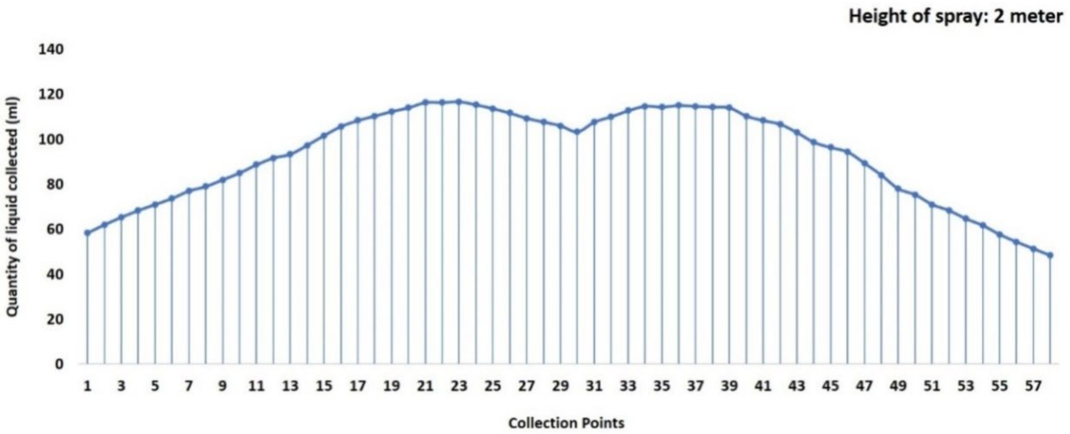

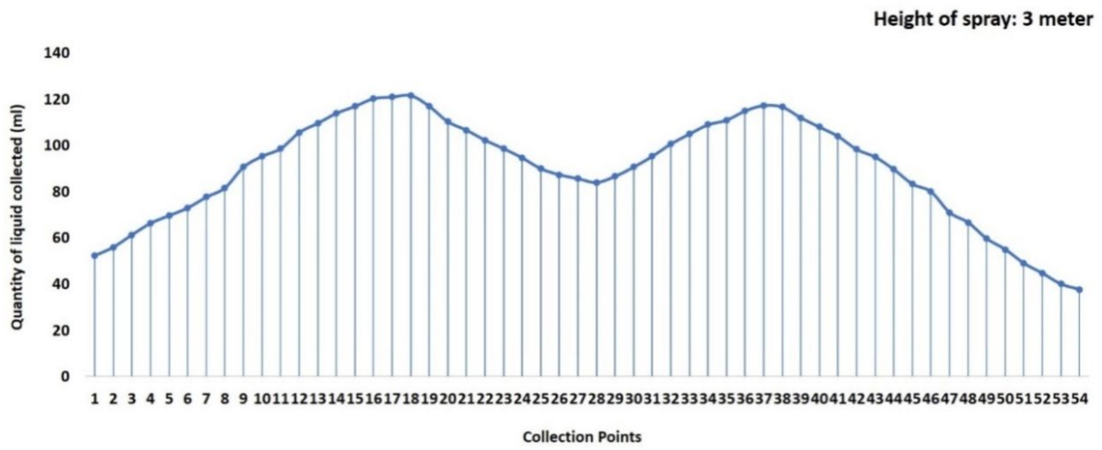

Supplementary Image 9: One-direction application spray volume distribution pattern of boom type nozzle configuration

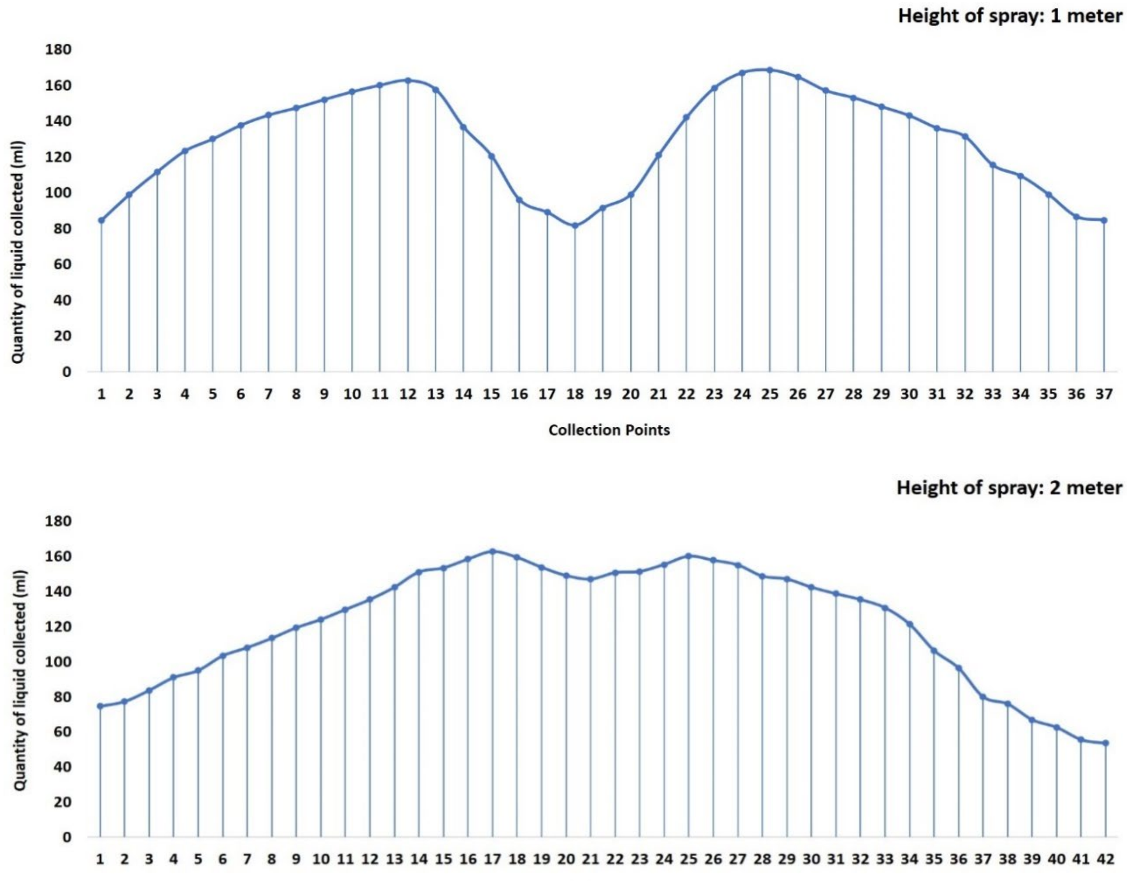

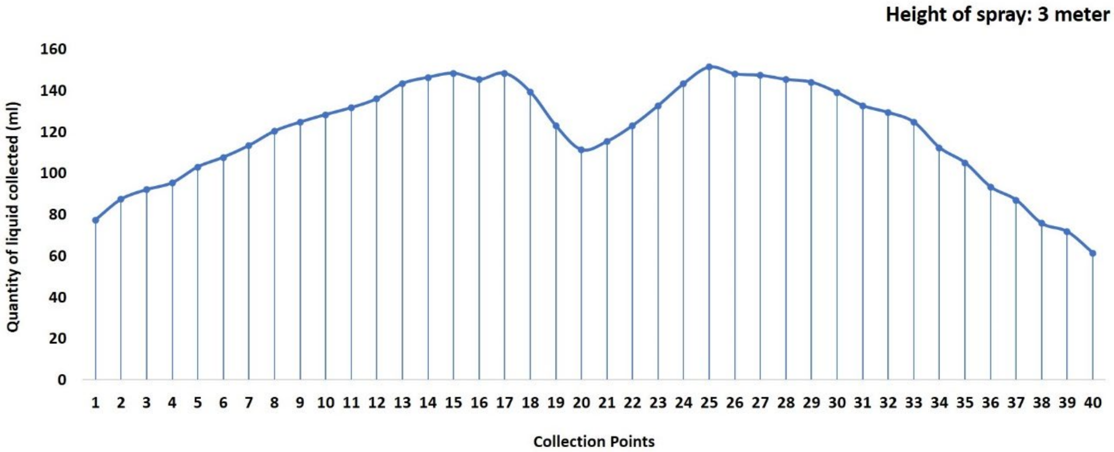

Supplementary Image 10: One-direction application spray volume distribution pattern of standard hexa type nozzle configuration
